# A clinically parameterized mathematical model of *Shigella* immunity to inform vaccine design

**DOI:** 10.1371/journal.pone.0189571

**Published:** 2018-01-05

**Authors:** Courtney L. Davis, Rezwanul Wahid, Franklin R. Toapanta, Jakub K. Simon, Marcelo B. Sztein

**Affiliations:** 1 Natural Science Division, Pepperdine University, Malibu, CA, United States of America; 2 Center for Vaccine Development, University of Maryland School of Medicine, Baltimore, MD, United States of America; 3 Merck & Co. Inc. Kenilworth, NJ, United States of America; Universitatsmedizin Greifswald, GERMANY

## Abstract

We refine and clinically parameterize a mathematical model of the humoral immune response against *Shigella*, a diarrheal bacteria that infects 80-165 million people and kills an estimated 600,000 people worldwide each year. Using Latin hypercube sampling and Monte Carlo simulations for parameter estimation, we fit our model to human immune data from two *Shigella* EcSf2a-2 vaccine trials and a rechallenge study in which antibody and B-cell responses against *Shigella′s* lipopolysaccharide (LPS) and O-membrane proteins (OMP) were recorded. The clinically grounded model is used to mathematically investigate which key immune mechanisms and bacterial targets confer immunity against *Shigella* and to predict which humoral immune components should be elicited to create a protective vaccine against *Shigella*. The model offers insight into why the EcSf2a-2 vaccine had low efficacy and demonstrates that at a group level a humoral immune response induced by EcSf2a-2 vaccine or wild-type challenge against *Shigella′s* LPS or OMP does not appear sufficient for protection. That is, the model predicts an uncontrolled infection of gut epithelial cells that is present across all best-fit model parameterizations when fit to EcSf2a-2 vaccine or wild-type challenge data. Using sensitivity analysis, we explore which model parameter values must be altered to prevent the destructive epithelial invasion by *Shigella* bacteria and identify four key parameter groups as potential vaccine targets or immune correlates: 1) the rate that *Shigella* migrates into the lamina propria or epithelium, 2) the rate that memory B cells (B_M_) differentiate into antibody-secreting cells (ASC), 3) the rate at which antibodies are produced by activated ASC, and 4) the *Shigella*-specific B_M_ carrying capacity. This paper underscores the need for a multifaceted approach in ongoing efforts to design an effective *Shigella* vaccine.

## Introduction

*Shigella*, a dysentery-causing bacterial pathogen in the same family as *Escherichia coli*, causes an estimated 80-165 million dysentery infections and kills an estimated 600,000 people worldwide every year, with the highest prevalence in developing countries [1, 2]. Treatment of shigellosis has become increasingly difficult with the spread of antibiotic resistance in *Shigella*. Furthermore, no vaccine has been licensed to prevent *Shigella* infections despite decades of clinical trials; difficulties in *Shigella* vaccine design include scarce, imperfect animal models and an incomplete understanding of how the immune system successfully neutralizes a *Shigella* infection. Identifying the immunological correlates of protection—the immune system components that either prevent or correlatively indicate the prevention of shigellosis symptoms—has been declared perhaps the most crucial step for accelerating vaccine design and yet continues to prove elusive [3, 4].

*Shigella* infections occur via fecal-oral transmission [2]. Upon ingestion of contaminated food or water, the bacteria travels to the gut and crosses from the lumen into the lamina propria (LP, just inside the gut lining) via host M cells; from the basolateral side of the epithelial lining, *Shigella* can enter and spread through epithelial cells and induce the most severe shigellosis symptoms [5–7]. This combination of intracellular and extracellular stages requires a multifaceted immune response for clearance after a *Shigella* infection has established. Clearance of intracellular bacteria likely requires the activation of cytotoxic T-cells and T-cell-derived cytokines, yet the role of cell-mediated immunity (CMI) in protection from *Shigella* is largely unknown [6, 8–16]. In this paper, we do not evaluate CMI responses and thus our model investigates the humoral, antibody-based effects of a *Shigella* vaccine with a goal not only to eradicate the bacteria from the gut lumen and lamina propria but also to prevent *Shigella* from entering epithelial cells. Vaccine targets in past *Shigella* vaccine candidates include lipopolysaccharide (LPS) and O-membrane proteins (OMP) on the bacterial surface as well as protein components of the bacteria’s epithelial entry machinery such as IpaB and MxiH [3, 17]. Studies suggest that serum immunoglobulin G (IgG) targeting LPS and antibody secreting cells (ASC) that secrete anti-LPS immunoglobulin A (IgA) correlate with *Shigella* protection [3, 6, 18–20]. However, whether anti-LPS antibodies or ASC can be sufficient for protection remains unknown, as does which immune factors paired with which bacterial targets induce the most effective anti-*Shigella* response. We use mathematical tools to help elucidate these key relationships.

Mathematical models of immune responses to viruses and some bacteria are increasingly common (e.g., [21–26]), and a few mathematical studies have examined in-host pathogen interactions with vaccines (e.g., [27, 28]). Our 2013 paper [29] introduced the first mathematical investigation of the immune response against *Shigella*; with it, we established a foundational mathematical framework for identifying key bacterial and immune vaccine targets [29]. That model used delay differential equations to capture the interactions between *Shigella* and the humoral immune response composed of antibodies (Ab), antibody-secreting cells (also known as plasma cells), and memory B cells (B_M_). One-day delays were incorporated into the equations to mimic the time delay from antigenic activation of naive B cells to the production of new ASC or B_M_. That first model was parameterized with rate values from the literature, as a more thorough parameterization with clinical data was beyond the scope of the paper. Our modeling results predicted that an anti-LPS vaccine would be ineffective at preventing shigellosis symptoms without a massive boost in antibody, ASC, and/or B_M_ numbers [29].

In this paper, we clinically parameterize our mathematical model by fitting the model to clinical human data from three *Shigella* vaccine and challenge trials in the 1990s. The model is fit to average immune levels within trial groups, and thus protection is evaluated on a group rather than individual level. We use Latin hypercube sampling, least squares optimization, and Monte Carlo simulations to explore the relevant multi-dimensional parameter space and to identify best-fit parameter value combinations. We also update our mathematical model introduced in [29] with key structural changes. Most notably, we use ordinary instead of delay differential equations for numerical efficiency while carrying out Monte Carlo runs during parameter estimation. We also directly include memory B cell stem-like activity (that is, intermittent asymmetric division of B_M_ to maintain the B_M_ pool) to counteract the degree of memory cell depletion as B_M_ differentiate into ASC that was observed with the first model, which assumed a baseline logistic B_M_ growth rate and then only purely symmetric differentiation of B_M_ into ASC. After comprehensive clinical parameterization, we perform identifiability analysis and use numerical sensitivity analysis to identify the few critical parameter values that govern the clearance and severity of *Shigella* infections. With these, we make model-based predictions about which biological interactions might—and which biological interactions likely do not—correlate with protective immunity against *Shigella*. We find that antibody-based vaccines targeting *Shigella′s* outer membrane components such as LPS and OMP are unlikely to elicit sufficient immunity for protection at a group level except possibly with major boosts in particular immune parameters, which we identify. Our modeling suggests secondary humoral immune targets upon which to focus in future vaccine development efforts. These include 1) the rate that *Shigella* migrates into the lamina propria or epithelium, 2) the rate that B_M_ differentiate into ASC, 3) the rate at which antibodies are produced by activated ASC, and 4) the *Shigella*-specific B_M_ carrying capacity (that is, the number of *Shigella*-specific B_M_ at homeostasis between infections). Future modeling and vaccine efforts could also examine the efficacy of CMI and other immune targets not directly measured in these clinical studies and thus not included in the present model.

## Methods

### Data

**Fig 1:**
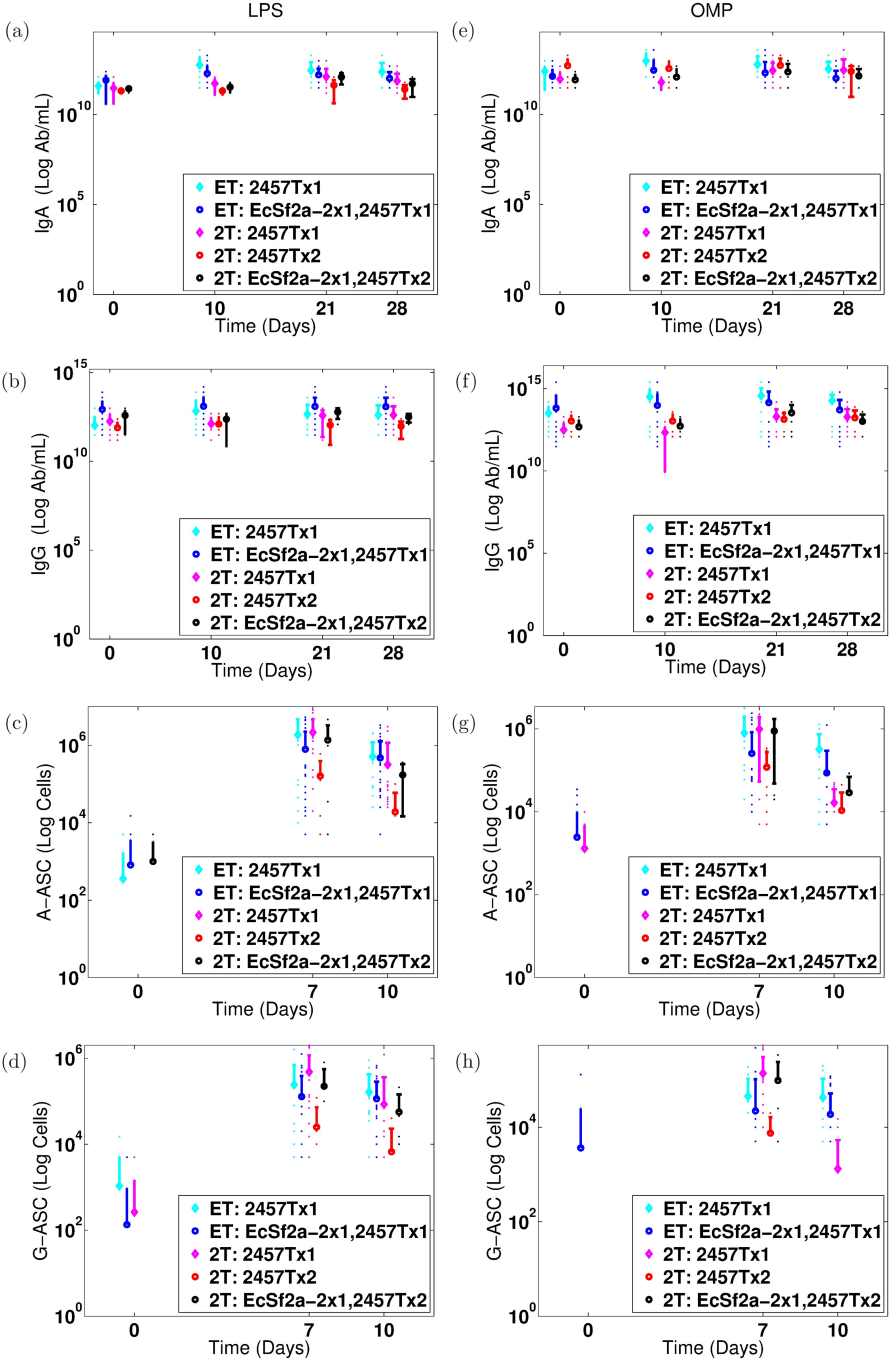
Data from EcSf2a-2 clinical trials and 2457T rechallenge study [30–32, 34]. Measured antibody (serum IgA, serum IgG), IgA-secreting ASC (A-ASC), and IgG-secreting ASC (G-ASC) directed against lipopolysaccharide (LPS, left) and O-membrane proteins (OMP, right) are given over time for each trial. We convert measured specific antibody titers to the number of specific Ab/mL by a factor of 1: 1.204 × 10^10^ Ab/mL, as described in the text. Measurements were taken 0, 10, 21, and 28 days after challenge for antibodies and on days 0, 7, and 10 for ASC; displays each day are offset to differentiate between trials. Dots represent each individual volunteer’s measured values. Means and standard deviations are plotted for each trial group. ET denotes data from the EcSf2a-2 vaccine trials whereas 2T denotes the 2457T rechallenge study. EcSf2a-2 indicates the vaccine was given, 2457T indicates wt challenge, and x1 or x2 indicates once or twice. The five trial groups are the following. **ET: 2457Tx1** (cyan): A primary immune response: controls from the EcSf2a-2 trials who were challenged with wild-type (2457T) *Shigella* without the vaccine. **ET: EcSf2a-2x1, 2457Tx1** (blue): An immune response following vaccination: all volunteers who received the EcSf2a-2 vaccine followed by wild-type challenge. **2T: 2457Tx1** (magenta): A primary immune response: unvaccinated volunteers for the 2457T rechallenge study who were challenged once with wild-type 2457T *Shigella*. **2T: 2457Tx2** (red): A wild-type secondary immune response: volunteers who first served as controls in EcSf2a-2 trials and were challenged with wild-type 2457T (but not vaccinated) in that study. These volunteers were then rechallenged with wild-type 2457T *Shigella* in the 2457T rechallenge study. **2T: EcSf2a-2x1, 2457Tx2** (black): A secondary immune response after vaccination: volunteers who received both the EcSf2a-2 vaccine and a 2457T challenge in the first studies. They then were rechallenged with wild-type *Shigella* in the 2457T rechallenge study. They have not been included in the model data fits.

We use clinical data supplied and published by our collaborators from three human clinical studies in the 1990s: two vaccine clinical trials followed by a rechallenge study [30–34]. The two vaccine trials (called the EcSf2a-2 studies in this paper or ET in labels) tested the efficacy of EcSf2a-2, a hybrid *E. coli-Shigella flexneri* 2a vaccine candidate [30–32]. This clinical trial separated volunteers into control and vaccinated groups, the latter of which were given the EcSf2a-2 vaccine. All volunteers were later challenged with 1,400 colony forming units (cfu) of 2457T wild-type (wt) *Shigella*. Volunteers were tracked for a month and time-course data were gathered at days 0, 7, and 10 for ASC of IgA- and IgG-type and at days 0, 14, 21, and 28 for antibodies—specifically, serum IgA and serum IgG directed at LPS and OMP in *Shigella′s* outer membrane. In the EcSf2a-2 vaccine studies, 14 volunteers received placebo while 45 volunteers received the vaccine in three or four doses. All volunteers then received the wild-type challenge with 2457T *Shigella*. We aggregate the respective volunteers in the EcSf2a-2 trials to form the placebo+wt primary infection data, which we label ET: 2457Tx1 for the EcSf2a-2 studies with no vaccine and one challenge of 2457T *Shigella*, and separately the vaccine+wt secondary infection data, which we label ET: EcSf2a-2x1, 2457Tx1 for the EcSf2a-2 trials with one round of EcSf2a-2 vaccine and then one challenge of 2457T vaccine. (One vaccine round includes all three or four doses, as applicable.) We fit our model against the mean ASC and antibody levels for each group. Fig 1 shows clinical measurements of antibodies and ASC targeting LPS (a–d) or OMP (e–h) during the challenge portion of the clinical trial. The trials demonstrated efficacy in 27-36% of volunteers but were not considered sufficiently efficacious on average at the group level.

In a follow-up to the EcSf2a-2 trials, 30 volunteers were challenged or rechallenged with 1,400 cfu of 2457T wt *Shigella*. We call this the 2457T study or 2T in labels. The 19 formerly immunologically naive individuals who received a 2457T challenge are labeled 2T: 2457Tx1, for the 2T study with one challenge of wt 2457T *Shigella*. Six other individuals, labeled 2T: 2457Tx2, had served as controls in the EcSf2a-2 study; these individuals had previously been challenged with 2457T *Shigella* without receiving the EcSf2a-2 vaccine candidate and in the second study were challenged a second time with wt 2457T *Shigella*. These data sets, also shown in Fig 1, thus show antibody and ASC levels directed against LPS and OMP both for formerly naive individuals after a primary infection and for previously exposed individuals after a secondary wild-type infection. In theory, the latter individuals should exhibit the reactivation of any natural immunity that was established during the primary infection against *Shigella*, although the level of immunity first established was not necessarily protective. A third category of 5 volunteers who received the EcSf2a-2 vaccine and challenge in the first trial followed by rechallenge in the second study (thus, possibly exhibiting tertiary immunity) are labeled 2T: EcSf2a-2x1, 2457Tx2 and are not included in this paper’s analysis to avoid mixing the effects of vaccine-induced versus wt-induced immunity.

Notice in Fig 1 that from primary to secondary immune responses there is in many cases no statistical rise and often slight declines in the levels of serum Ab and ASC directed against LPS and OMP. That is, the primary EcSf2a-2 (cyan) and primary 2457T (magenta) study data from controls who were challenged with wt *Shigella* are equal to or higher than the corresponding secondary or tertiary exposure data (cyan versus blue, magenta versus red and black) in many cases. We compare modeling results from parameters fit to the primary infection data, which predict theoretical increases in secondary immunity, to those fit to both primary and secondary infection data, which replicate this observed flatness in data. These separate fits enable us to gain insight into the potential effects of establishing LPS and/or OMP immunity at or above measured clinical values and also ensure the robustness of our conclusions across different parameterizations.

It is not known clinically what Ab and/or ASC levels are sufficient for immunity, if any, nor which targets are most immunologically active or involved in protection from disease [3, 35]. However, the 27-36% efficacy of the EcSf2a-2 trials indicates that measured levels of ASC, serum IgA, and serum IgG directed against LPS and OMP are not sufficient on average at the group level to prevent infection in the majority of patients. On the other hand, five of six volunteers who received a second wild-type dose of 2457T in the challenge study (that is, 2T: 2457Tx2) were not strongly symptomatic. It is not known if the mechanisms of protection were the measured levels of anti-LPS and anti-OMP serum IgA and/or IgG or other unmeasured immune components perhaps with different bacterial targets. With this mathematical model, we investigate this by using only the measured serum IgA and IgG humoral responses without any additional immune action and examine *in silico* whether epithelial infection is sufficiently prevented (indicating hosts with few-to-no symptoms).

To convert clinical data to units more readily usable in the mathematical model, we assume the following. To convert ASC and B_M_ per million peripheral blood mononuclear cells (PBMC) to absolute numbers of cells, we note that there are roughly 10^6^ PBMC per mL of whole blood [36] and roughly 5,000 mL of blood in a human body [37]). Thus, we multiply measured ASC or B_M_ cells per million PBMC by a factor of 5,000 to obtain an estimate of cell numbers. We do not have any established standard method to convert our serum IgA and IgG titers to number of antibodies per mL. Thus, we use the following approximation from titer to antibodies per milliliter (Ab/mL) where Ab here is the number of antibodies. IgA and IgG each have a molecular weight of 150 kDa (kg/mol) per Ab [37]. In our data, the measurement of the specific antibodies was quantified and expressed as calculated endpoint titers. However, using a parallel standard curve with known concentration of purified IgG or IgA in the same setup, we approximate that a titer of 1 approximately represents 3 ng/mL of antibody (unpublished data). Taking these factors together with Avogadro’s number, we convert our specific antibody titers to the number of specific Ab/mL by a factor of 1: 1.204 × 10^10^ Ab/mL.

### Mathematical model

**Fig 2:**
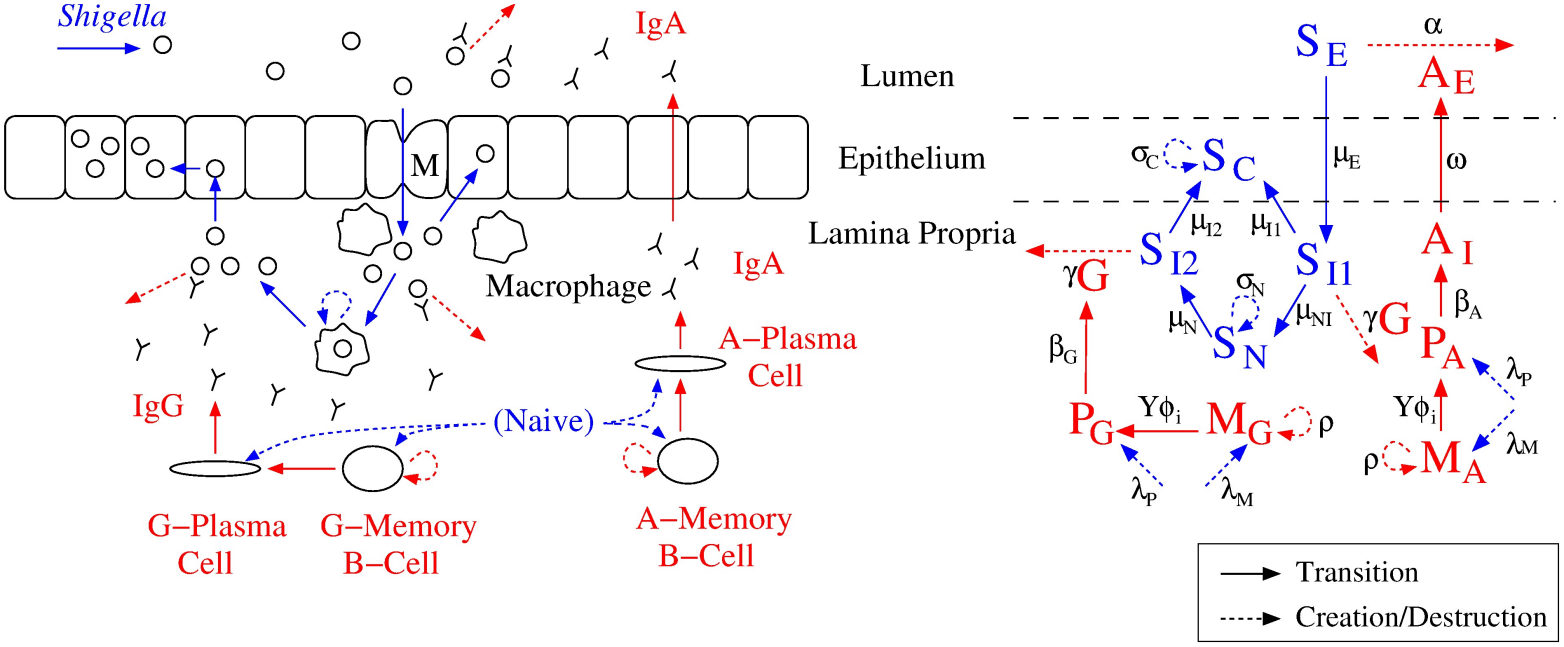
Key interactions between *Shigella* and the host’s humoral immune system in the gut. Bacterial pathogenesis (blue) plus antibody and B cell dynamics (red) seen in vivo (left) are translated to a mathematical reaction diagram (right). The most severe symptoms result when *Shigella* infects epithelial cells. Prior to this, *Shigella* can be removed by antibodies (lumen IgA or lamina propria IgG targeting *Shigella′s* outer membrane) or engulfed by macrophages, from which it escapes or is destroyed. All modeled components also degrade naturally; degradation arrows have been omitted for simplicity. Mathematical abbreviations: *S*: *Shigella*, *A*: IgA, *G*: IgG, *M*: B_M_, *P*: ASC, *I*: in Lamina Propria, *E*: Luminal, *C*: Epithelial, *N*: Engulfed.

We update our original model in [29] to a system of 12 ordinary differential equations that capture interactions between bacterial and humoral immune dynamics. Biological and mathematical reaction diagrams corresponding to the updated model are given in Fig 2. Model parameter definitions are listed in Table 1. The model focuses on humoral immune agents, specifically Ab, ASC, and B_M_. We include IgG- and IgA-type immunity for all Ab, ASC, and B_M_. For simplicity we do not include immunoglobulin M (IgM) production or affinity maturation/somatic hypermutation prior to IgA/IgG secretion, although we consider somatic hypermutation during interpretation of our sensitivity analysis results. We also do not include potential contributions from cytotoxic T-cells. While cell-mediated immunity could help to combat intracellular *Shigella* within the epithelium, the exact role and importance of effector cell-mediated activity in fighting *Shigella* infections remains to be well characterized; thus, any mathematical modeling predictions regarding CMI activity against *Shigella* would be difficult to experimentally corroborate or implement at this time [3].

**Table 1:** Parameter definitions.

Param	Range, Units	Description	Reference, Note
*σ*_*N*_	(0.01,1)/d	*Shigella* reproduction rate in innate immune cells	high but brief [38]
*σ*_*C*_	0.1/d	*Shigella* reproduction rate in epithelial cells	high [39, 40] (smaller for numerical stability w/o CTLs)
*δ*_*SE*_	0.0001/d	Natural death rate of *Shigella* in lumen	minimal
*δ*_*SI*_	0.0001/d	Natural death rate of *Shigella* in LP	minimal
*δ*_*SN*_	(0.1,1)/d	Death rate of *Shigella* in macrophages	1 − *μ*_*N*_
*δ*_*SC*_	0.0001/d	Natural death rate of *Shigella* in epithelium	minimal
*δ*_*AE*_	(0.01,0.5)/d	Natural decay/removal rate of IgA in lumen	3.5 d hl (from *δ*_*G*_)
*δ*_*AI*_	(0.01,0.5)/d	Natural decay/removal rate of IgA in LP	3.5 d hl (from *δ*_*G*_)
*δ*_*G*_	(0.01,0.5)/d	Natural decay/removal rate of IgG in LP	3.5 d hl (18 d hl [41]-localiz.)
*δ*_*PA*_	0.2/d	Natural death rate of IgA- type ASC	3 d hl (early pc) [41–43]
*δ*_*PG*_	0.2/d	Natural death rate of IgG-type ASC	3 d hl (early pc) [41–43]
*μ*_*E*_	(0.1,1)/d	Migration rate of *Shigella* from lumen to LP	low (0.0029% of 10^8^ bact/mL) in first 2h [44]
*μ*_*I*_	(0.1,1)/d	Migration rate of *Shigella* from LP to epithelium	
*μ*_*NI*_	(0.5,0.9)/d	Rate *Shigella* in LP are engulfed by macrophages	fast
*μ*_*N*_	(0.5,0.9)/d	Rate *Shigella* escape out of macrophages into LP	fast (macs apoptose in 8h) [38]
*ω*	(0.01,1)/d	Migration rate of IgA from LP to lumen	20% of total IgA to lumen (from *n*_*I*_)
*n*_*E*_	(2e3,2e4) Ab/mL/cfu	Number of IgA that neutralize a luminal bacterium	
*n*_*I*_	(2e3,2e4) Ab/mL/cfu	Number of IgG that neutralize a LP bacterium	[45]
*β*_*A*_	(5e4,5e7) (Ab/mL)/pc/d	Production rate of IgA by IgA-type ASC	10^5^/sec [41, 46–49]
*β*_*G*_	(5e4,5e7) (Ab/mL)/pc/d	Production rate of IgG by IgG-type ASC	10^5^/sec [41, 46–49]
*ϕ*_1_	(0.001,0.1)/d	Antigen-independent differentiation rate of B_M_ into ASC	
*ρ*	(0.001,0.1)/d	Antigen-independent cycling rate of B_M_	
*ξ*_1_	(1,1e4) pc/mc	Number of ASC generated by background B_M_ proliferation	
*ξ*_2_	(5e2,5e4) pc/mc	Number of ASC generated by proliferating antigen-activated B_M_	
*α*	(1e-16,1e-13)/(Ab/mL)/d	Rate antibodies neutralize *Shigella* in lumen	
*γ*	(1e-16,1e-13)/(Ab/mL)/d	Rate antibodies neutralize *Shigella* in LP	
*ϕ*_2*A*_	(0.001,1)/cfu/d	Antigen-dependent rate function for IgA-type B_M_ differentiation into ASC	
*ϕ*_2*G*_	(0.001,1)/cfu/d	Antigen-dependent rate function for IgG-type B_M_ differentiation into ASC	
*k*_*A*_	(5e2,5e5) mc	IgA-type B_M_ carrying capacity	
*k*_*G*_	(5e2,5e5) mc	IgG-type B_M_ carrying capacity	
*λ*_*P*_	(1e-2,1e4) pc/cfu/d	Creation rate of new ASC from naive B cells	All *λ* are set equal.
*λ*_*M*_	(1e-2,1e4) mc/cfu/d	Creation rate of new B_M_ from naive B cells	
Υ	(0.05,1)	Percent of B_M_ differentiations that do not create new B_M_ via stem-like activity	
Delay	3.5 d	Initial delay until naive B cell activation in an infection	
*S*_*E*_(0)	1,400 cfu	Initial number of luminal *Shigella*	Clinical trials [30–32, 34]

Parameters of the model are given along with their descriptions and ranges for Latin hypercube sampling. Parameters with fixed numbers rather than ranges are kept constant and not fit. Applicable references or notes on the range values are given. Ranges for uncited parameters are chosen to be broad, subject to plausibility and numerical stability. The initial condition for establishing a (luminal) *Shigella* infection is also given. Other initial conditions are taken from the clinical studies or calculated disease-free equilibria. Abbreviations: d: day, Ab: antibodies, mL: milliliter, cfu: colony-forming units, pc: plasma cells (ASC), mc: B_M_ cells, hl: half-life, LP: lamina propria.

Our 2013 model [29] used delay differential equations to capture the one-day time delay from naive B cell activation to the production of functional ASC or B_M_. However, numerical discretization and simulation of these delay differential equations are slow and often stiff due to wide biologically relevant parameter ranges; for numerical efficiency during Monte Carlo simulations for parameter estimation and data fitting, we eliminate the delays and instead use ordinary differential equations. This decision is supported by sensitivity analysis of our original delay model [29], which suggested that these delays have little effect on peak bacterial or antibody magnitudes. By using the best-fit parameter tuples from clinical parameterization of this paper’s updated non-delay model to parameterize and simulate a delay differential equation version of our model, we have confirmed that the one-day delayed model produces visually identical simulations to those in this paper (not shown). Despite eliminating these built-in naive cell activation delays, we continue to externally numerically enforce an initial 3.5-day delay after infection begins during which bacteria and any previously established immunity interact without yet eliciting new antigen-induced B cell formation. This time window does not affect pre-existing immunity or innate immune cells such as macrophages, which are modeled indirectly via the engulfed *Shigella* population.

Our model tracks bacterial populations in the gut lumen, epithelium, and lamina propria after ingestion of contaminated food or water. As an infection begins, *Shigella* in the lumen, denoted *S*_*E*_, crosses the epithelial barrier at rate *μ*_*E*_ via the normal activity of host M cells, which shuttle material from the lumen across the epithelium to the LP. Upon reaching the LP, *Shigella*, now *S*_*I*1_, is often engulfed at rate *μ*_*NI*_ by nearby innate immune cells such as macrophages that serve as a first line of host defense by engulfing and destroying invaders. However, engulfed *Shigella*, *S*_*N*_, typically avoids destruction and instead proliferates at rate *σ*_*N*_ inside of macrophages. While inside, *Shigella* is subject to a small rate *δ*_*SN*_ of macrophage killing but is safe from antibody targeting. After *Shigella* breaks out of the macrophage at rate *μ*_*N*_, becoming *S*_*I*2_, we assume the bacterium is sufficiently distant from M cells and other macrophages to have no likelihood of re-engulfment. We noted in [29] that separate *S*_*I*1_ and *S*_*I*2_ LP populations are mathematically necessary to prevent macrophages from becoming a chronic reservoir for *Shigella* infections. However, either bacterial population can infect epithelial cells at rate *μ*_*I*_, and once inside, they survive as *S*_*C*_, proliferate at rate *σ*_*C*_, and move freely between epithelial cells without interference from innate or humoral immune defenses. Substantial epithelial cell destruction due to this cellular invasion is primarily responsible for inducing the most severe symptoms of shigellosis [50].

We summarize the bacterial dynamics with the following equations.

$$\begin{array}{ccc} \frac{dS_{E}}{dt} & = & -\delta_{SE}S_{E}-\mu_{E}S_{E}-\alpha A_{E}S_{E} \end{array}$$ (1)

$$\begin{array}{ccc} \frac{dS_{I1}}{dt} & = & -\delta_{SI}S_{I1}+\mu_{E}S_{E}-\mu_{NI}S_{I1}-\mu_{I}S_{I1}-\gamma GS_{I1} \end{array}$$ (2)

$$\begin{array}{ccc} \frac{dS_{N}}{dt} & = & \sigma_{N}S_{N}-\delta_{SN}S_{N}+\mu_{NI}S_{I1}-\mu_{N}S_{N} \end{array}$$ (3)

$$\begin{array}{ccc} \frac{dS_{I2}}{dt} & = & -\delta_{SI}S_{I2}-\mu_{I}S_{I2}+\mu_{N}S_{N}-\gamma GS_{I2} \end{array}$$ (4)

$$\begin{array}{ccc} \frac{dS_{C}}{dt} & = & \sigma_{C}S_{C}-\delta_{SC}S_{C}+\mu_{I}\left(S_{I1}+S_{I2}\right) \end{array}$$ (5)

The *δ* terms account for death due to natural causes, spatial washout, or macrophage activity; *Shigella* removal via antibody is modeled separately.

Interactions between bacteria and the immune system occur when *Shigella* becomes bound by IgA in the lumen (written *A*_*E*_) or IgG in the LP (denoted *G*) at rates *α* and *γ*, respectively. Although ASC initially produce IgM before somatically hypermutating and class switching to produce IgA and IgG, for simplicity our model only includes IgA, the most abundant and active antibody isotype at mucosal surfaces, and IgG, the most abundant antibody in serum [37, 51–54]. We focus on IgA and IgG antibodies that recognize and bind specifically to *Shigella* outer membrane components, such as LPS or OMP, that are continuously displayed and hence subject to antibody targeting whenever *Shigella* exists outside of host cells. Equations governing antibody dynamics can be found below. When sufficient numbers (*n*_*E*_ or *n*_*I*_, respectively) of IgA or IgG bind to a *Shigella* bacterium, that bacterium is removed or destroyed. We use mass action interaction terms as a null model to describe bacterial interaction with and removal via antibodies; more complex functional forms are not necessary to match our clinical data. Adding saturating interaction terms would slow immune control of *Shigella* as antibodies approach a maximum capacity for bacterial neutralization; in comparison, model bacterial dynamics as written could underestimate *Shigella* numbers and may be a best-case scenario.

The IgG antibody response to *Shigella* in the LP is largely mediated by IgG-secreting ASC (plasma cells), *P*_*G*_, and upstream IgG-type memory B cells, *M*_*G*_. Similarly, IgA antibodies are created in the LP by IgA-secreting ASC, *P*_*A*_, which can be derived from IgA-type B_M_ cells, *M*_*A*_, or naive B cells (not explicitly modeled); IgA in the LP, *A*_*I*_, then shuttles across the epithelial barrier at rate *ω* to the lumen where IgA primarily functions, becoming *A*_*E*_. Irrespective of infection state, B_M_ differentiate at baseline rate *ϕ*_1_ to produce *ξ*_1_ ASC, which constantly secrete antibodies at rate *β*_*A*_ or *β*_*G*_. However, the presence of bacteria within the lamina propria stimulates naive B cells to create new ASC at rate *λ*_*P*_ and new B_M_ cells at rate *λ*_*M*_ while also antigenically stimulating additional B_M_ differentiation to create *ξ*_2_ new ASC at rate *ϕ*_2*A*_ or *ϕ*_2*G*_. The creation of ASC from B_M_ is lessened slightly by *Υ* and Υ* as described below.

These immune dynamics are captured with the following model equations, which together with the bacterial dynamics constitute the full mathematical model.

$$\begin{array}{ccc} \frac{dA_{E}}{dt} & = & -\delta_{AE}A_{E}+\omega A_{I}-n_{E}\alpha A_{E}S_{E} \end{array}$$ (6)

$$\begin{array}{ccc} \frac{dA_{I}}{dt} & = & -\delta_{AI}A_{I}-\omega A_{I}+\beta_{A}P_{A} \end{array}$$ (7)

$$\begin{array}{ccc} \frac{dG}{dt} & = & -\delta_{G}G+\beta_{G}P_{G}-n_{I}\gamma G\left(S_{I1}+S_{I2}\right) \end{array}$$ (8)

$$\begin{array}{ccc} \frac{dP_{A}}{dt} & = & -\delta_{PA}P_{A}+\lambda_{P}\left(S_{I1}+S_{I2}\right)+{ϒ}^{*}(\xi_{2}\phi_{2A}\left(S_{I1}+S_{I2}\right)+\xi_{1}\phi_{1})M_{A} \end{array}$$ (9)

$$\begin{array}{ccc} \frac{dP_{G}}{dt} & = & -\delta_{PG}P_{G}+\lambda_{P}\left(S_{I1}+S_{I2}\right)+{ϒ}^{*}(\xi_{2}\phi_{2G}\left(S_{I1}+S_{I2}\right)+\xi_{1}\phi_{1})M_{G} \end{array}$$ (10)

$$\begin{array}{ccc} \frac{dM_{A}}{dt} & = & \rho (1-\frac{M_{A}}{k_{A}})M_{A}+\lambda_{M}\left(S_{I1}+S_{I2}\right)-ϒ(\phi_{2A}\left(S_{I1}+S_{I2}\right)+\phi_{1})M_{A} \end{array}$$ (11)

$$\begin{array}{ccc} \frac{dM_{G}}{dt} & = & \rho (1-\frac{M_{G}}{k_{G}})M_{G}+\lambda_{M}\left(S_{I1}+S_{I2}\right)-ϒ(\phi_{2G}\left(S_{I1}+S_{I2}\right)+\phi_{1})M_{G} \end{array}$$ (12)

In the absence of an infection, B_M_ cell generation, death, and differentiation rates must balance to establish an equilibrated immune state with a steady nontrivial (nonzero) memory population. To prevent the mathematical system from becoming neutrally stable at this equilibrium, we assume B_M_ follow logistic growth dynamics with growth rate *ρ* and carrying capacity *κ*_*A*_ or *κ*_*G*_. The carrying capacity is the maximum sustainable population of antigen-specific B_M_; that is, it is the size of the *Shigella*-specific B_M_ population at homeostasis between infections.

**Fig 3:**
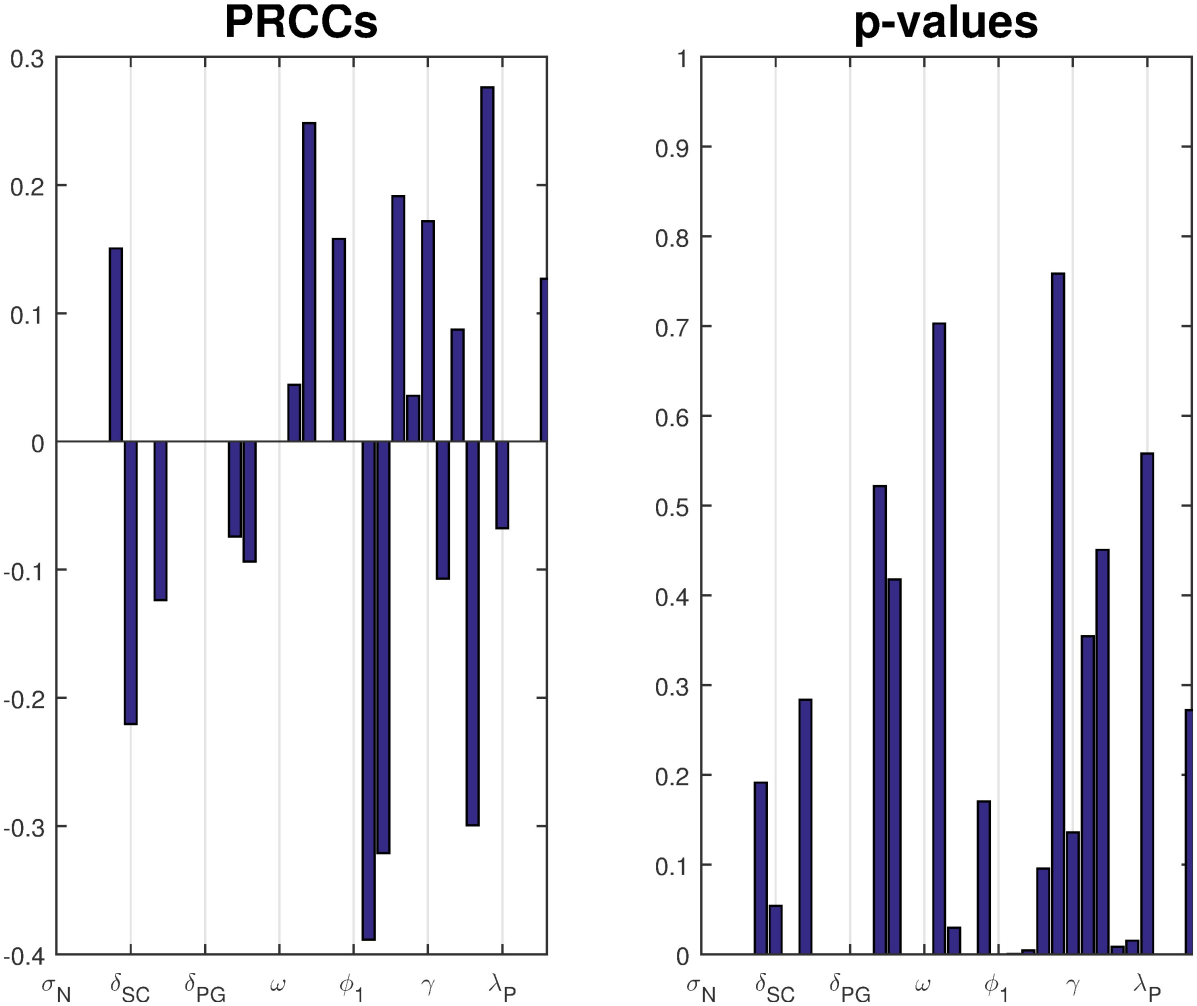
Partial Rank Correlation Coefficients (PRCCs) for all best-fit runs fit to both primary and secondary infection data calculated together along with their p-values. Parameters are listed on the horizontal axis in the same order as Table 1. Extreme PRCC values (> 0.5 or < −0.5) and small p-values indicate parameters that are key for how well the model fits to data. Since all parameters have PRCC values closer to 0 than to ±1, no parameters stand out as most important for these fits. Despite the small PRCC values, some parameters have a p-value less than 0.05: *μ*_*I*_, *ξ*_2_, *ϕ*_1_, *ϕ*_2*A*_, *ϕ*_2*G*_; thus, these parameters could have more influence than others in fitting the model to data. Importantly, the parameters most responsible for fitting the model to data could be different than the parameters most responsible for preventing an epithelial infection and resulting symptoms in a host; the latter is explored separately with the sensitivity analysis results.

In our 2013 paper [29], we found that raising the B_M_ carrying capacity could greatly reduce bacterial load and thus suggested that the B_M_ carrying capacity be investigated further as a potential correlate of *Shigella* immunity. If B_M_ numbers are a limiting factor in establishing *Shigella* protective immunity, as indicated by the near-depletion of the B_M_ population in Figs 2 and 3 of that paper [29], sustained B_M_ levels might indeed be crucial. However, the observed depletion could be an artifact of the modeling assumption that B_M_ symmetrically divide and differentiate to form ASC if such differentiation outpaces the assumed logistic growth of B_M_. Since B_M_ also sometimes act as stem-like cells and divide to replenish their own population [55–57], we update the model to include this mixed multiplication strategy. Specifically, all memory differentiation rates are modulated by the percentage *Υ* or Υ*, which are defined as follows. We assume that B_M_ can symmetrically differentiate to produce *ξ*_*i*_ ASC (where *i* = 1 for antigen-independent differentiation and *i* = 2 for antigen-dependent differentiation) and that this symmetric differentiation occurs *Υ*% (written as a decimal) of memory differentiations. The remaining 1 − *Υ*% of differentiations involve a B_M_ asymmetrically dividing to produce one B_M_ replacement and a plasma cell progenitor that immediately differentiates and divides to produce half as many (that is, *ξ*_*i*_/2) ASC. The temporary progenitor cell is not explicitly modeled. Hence, overall a total of *Υ*% of old B_M_ are lost at the rates given in the model as a total of Υ*ξ_*i*_ ASC per B_M_ are gained, where

$${ϒ}^{*}=(\frac{1-ϒ}{2}+ϒ).$$

The degree of depletion observed might depend upon the balance between differentiation and self-replication established by *Υ*, and thus we examine the robustness of modeling results to a range of values for this parameter.

Because we do not examine cytotoxic T-cell interactions with intracellular *Shigella*, we also do not model cell-to-cell spread or epithelial cell destruction directly but rather assume high values for *S*_*C*_ indicate substantial epithelial cell destruction and severe illness [50]. We also limit our scope to within-host dynamics and do not model any potential bacterial migration from the epithelium back to the lumen (*S*_*C*_ → *S*_*E*_) before transmitting via a fecal-oral route to another person.

### Equilibrium analysis

Bacteria inside the epithelium, *S*_*C*_, are safe from humoral immune targeting and, if present, self-perpetuate and grow without bound in the model, even while the rest of the system reaches equilibrium; a goal of vaccination is to prevent this population from establishing. For equilibrium analysis we leave any growing *S*_*C*_ population out through quadrature. The remaining system of equations yields a trivial equilibrium as well as three nontrivial disease-free equilibria: IgA-type only, IgG-type only, and one with both IgA- and IgG-type Ab, ASC, and B_M_. Nontrivial equilibria represent states in which some level of immunity has been established, although perhaps not at protective levels. We assume both IgA and IgG immune responses are solicited and thus concentrate on the joint IgA/IgG nontrivial equilibrium, for which the equations and parameterized values are given in Table 2.

**Table 2:** Nontrivial disease-free equilibrium values.

	*S*_*E*_, *S*_*I*1_, *S*_*N*_, *S*_*I*2_, *S*_*C*_	*A*_*E*_	*A*_*I*_	*G*	*P*_*A*_, *P*_*G*_	*M*_*A*_, *M*_*G*_
	cfu	Ab/mL	Ab/mL	Ab/mL	Cells	Cells
EcSf2a-2 LPS Study	0	3.1 × 10^9^	3.5 × 10^9^	2.4 × 10^12^	9,610	20,050
Fit Primary Data					75,146	156,780
2457T LPS Study	0	5.1 × 10^10^	1.2 × 10^11^	5.6 × 10^11^	15,685	19,957
Fit Primary Data					7,647	9,730
EcSf2a-2 OMP Study	0	3.1 × 10^9^	3.5 × 10^9^	2.4 × 10^12^	9,610	20,050
Fit Primary Data					75,146	156,780
2457T OMP Study	0	2.1 × 10^9^	4.7 × 10^8^	2.7 × 10^11^	24	933
Fit Primary Data					69	2,668
EcSf2a-2 LPS Study	0	6.0 × 10^10^	1.4 × 10^11^	2.2 × 10^11^	611,620	88,311
Fit Primary and Secondary Data					3,040	439
2457T LPS Study	0	2.0 × 10^7^	1.3 × 10^7^	1.6 × 10^8^	4	73
Fit Primary and Secondary Data					4	71
EcSf2a-2 OMP Study	0	1.8 × 10^11^	3.5 × 10^10^	1.7 × 10^13^	75,926	8,585
Fit Primary and Secondary Data					196,870	22,261
$\begin{array}{lllllll} \text{Nonzero}\text{Terms} & A_{E}= & \frac{\omega }{\delta_{AE}}A_{I} & P_{A}= & \frac{\Upsilon^{*}\xi_{1}\phi_{1}}{\delta_{PA}}M_{A} & M_{A}= & \frac{\kappa_{A}(\rho -\Upsilon \phi_{1})}{\rho }\\ & A_{I}= & \frac{\beta_{A}}{\delta_{AI}+\omega }P_{A} & P_{G}= & \frac{\Upsilon^{*}\xi_{1}\phi_{1}}{\delta_{PG}}M_{G} & M_{G}= & \frac{\kappa_{G}(\rho -\Upsilon \phi_{1})}{\rho }\\ & G= & \frac{\beta_{G}}{\delta_{G}}P_{G} & & & & \end{array}$						
$\begin{array}{llll} \text{Eigenvalues}\;\text{of}\;\text{Jacobian} & -\delta_{PG} & \sigma_{N}-\delta_{SN}-\mu_{N} & -\delta_{SI}-\mu_{I}-\frac{\gamma \beta_{G}\xi_{1}\phi_{1}\kappa_{G}}{\delta_{G}\delta_{PG}\rho }\Upsilon^{*}(\rho -\Upsilon \phi_{1})\\ \text{at}\;\text{Nontrivial}\;\text{Equilibrium} & -\delta_{G} & -(\rho -\Upsilon \phi_{1}) & -\delta_{SI}-\mu_{I}-\mu_{NI}-\frac{\gamma \beta_{G}\xi_{1}\phi_{1}\kappa_{G}}{\delta_{G}\delta_{PG}\rho }\Upsilon^{*}(\rho -\Upsilon \phi_{1})\\ & \delta_{PA} & -(\rho -\Upsilon \phi_{1}) & -\delta_{SE}-\mu_{E}-\frac{\alpha \omega \beta_{A}\xi_{1}\phi_{1}\kappa_{A}}{\delta_{A}E(\delta_{AI}+\omega )\delta_{PA}\rho }\Upsilon^{*}(\rho -\Upsilon \phi_{1})\\ & \delta_{AE} & \delta_{AI}-\omega & \end{array}$						

Values of model state variables at the nontrivial disease-free equilibrium for which both IgA and IgG are present are given for the EcSf2a-2 trials or 2457T rechallenge study data. For each, the model is parameterized with data fit to either primary infection data alone or to both primary and secondary infection data. The positive, stable nontrivial equilibrium values are given, representing the long-term presence of some immunity, while in the unstable cases, the trivial equilibrium is instead approached. No positive nontrivial equilibrium was found for parameters that best fit the OMP measurements for the 2457T rechallenge study. The nontrivial equilibrium is evaluated at the corresponding parameter values given in the other tables. The equations for this nontrivial equilibrium are also given, as well as eigenvalues of the Jacobian for the linearized model. The equilibria are all positive and the eigenvalues are all negative if the positivity and stability conditions given in the text are met. These are sufficient, and for positivity necessary, conditions.

A positive, stable nontrivial equilibrium is necessary, although perhaps not sufficient, for establishing immunity to *Shigella* via vaccine or natural infection. Positivity and stability conditions for maintenance of such a nontrivial steady state can be derived by examining the eigenvalues of the Jacobian matrix evaluated at the joint IgA/IgG nontrivial equilibrium. These eigenvalues are given in Table 2. Positivity of the nontrivial equilibrium requires

$$\begin{array}{ccc} \rho -ϒ\phi_{1} & > & 0\text{,} \end{array}$$ (13)

which essentially requires that there be a balance between B_M_ logistic growth and differentiation into ASC; that is, B_M_ must not fully deplete. Stability requires the same condition as positivity plus that

$$\begin{array}{ccc} \sigma_{N} & < & \delta_{SN}+\mu_{N}\text{.} \end{array}$$ (14)

This requires that the bacterial growth rate inside of macrophages be less than the rate of bacterial exit so that a chronic or uncontrolled bacterial reservoir is not created inside of macrophages. If stability conditions are satisfied, the nontrivial equilibrium (some immunity) is approached as a stable sink while the trivial equilibrium (no immunity) is a saddle, as are the IgA-only and IgG-only nontrivial equilibria. There is a transcritical bifurcation (not shown) at which the nontrivial IgA/IgG equilibrium becomes negative and switches stability with the trivial equilibrium.

### Model implementation

A necessary condition for long-term immunity is a stable nontrivial disease-free equilibrium in the mathematical model. Because the biological threshold for sufficient, i.e. protective, immunity against *Shigella* is unknown, we explore the protective effects of this nontrivial immune state via our model. First, however, we find parameter value combinations that best fit the model to our clinical data using Latin hypercube sampling and a least squares metric as described below. In the process, we also uncover separate parameter combinations that ensure versus negate stability of the nontrivial immune state; that is, some parameter combinations enable some long-term immunity to establish while other combinations establish no long-term immunity. We numerically investigate the infection dynamics that lead to each. Finally, we use numerical sensitivity analysis to determine which parameter values do and do not control bacterial clearance and the establishment of lasting immunity.

We numerically implement infection dynamics in Matlab using a stiff ODE solver (ode15s). Both primary and secondary infections are simulated so that disease progression from nonimmune versus immune initial states can be compared. We initialize the model using as our initial luminal bacterial level, *S*_*E*_, the *Shigella* wt or vaccine single-dose levels administered in the clinical trials and reinfections studies: 2 × 10^9^ cfu for the EcSf2a-2 vaccine and 1,400 cfu for any wt challenges [30–32, 34]. Both are above the minimum infectious dose of 100-1,000 cfu [20, 31, 32, 35]. A primary infection is initialized with all other immune and bacterial numbers initially at 0. If a primary infection fails to establish any lasting immunity, as indicated by an unstable nontrivial equilibrium for a given parameterization, we initialize a secondary infection identically to the primary infection. If, on the other hand, the nontrivial IgA/IgG disease-free equilibrium is stable (with some immunity established), we initialize the secondary infection with immune numbers set to this disease-free equilibrium and then pulse 1,400 cfu of luminal *Shigella* to start the new infection. Thus, our model initialization mimics the *Shigella* challenges administered during the clinical studies.

In order to numerically enforce an initial 3.5-day incubation period after infection begins during which bacteria and any previously established immunity interact without yet eliciting new antigen-induced B cell formation, the mathematical model’s terms for antigen-dependent B cell proliferation and differentation are tagged and assigned the value zero for the prescribed length of time after initialization of the model simulation, after which these terms switch on to their nonzero values. Such an activation window has no effect on long-term, equilibrium immune levels because bacterial levels are zero at all equilibria and hence there is no chronic antigen induction of immunity. We also investigate how the existence and length of this activation window affect key short-term dynamics and find that, for instance, epithelial *Shigella* (*S*_*C*_) levels vary only slightly for between a 0- and 10-day activation delay (not shown).

### Parameter estimation

As described earlier, we have serum IgA, serum IgG, IgA-type ASC, and IgG-type ASC time series data from both primary and secondary infections. We focus separately on those that target two bacterial outer membrane components, LPS or OMP, in three different studies, the two EcSf2a-2 vaccine studies and the wild-type *Shigella flexneri* 2457T challenge study. For the EcSf2a-2 versus 2457T studies separately and for each target separately, our parameter estimations simultaneously fit the model to serum IgA, serum IgG, and both ASC types. It is important to note IgA was not measured in the gut lumen (i.e., stool) of subjects in these studies, and serum IgA is used as an indirect estimate of luminal IgA. We ignore time 0 data points in these fits, as initial measurements cannot always detect low levels of immune components, but we include all other time points. We initially fit only the primary infection with the hope to use the secondary infection data for independent model validation. However, since primary and secondary data from these clinical trials are very similar, as can be seen in Fig 1, the rise in secondary immunity predicted by primary fits does not often match clinical trends in secondary responses. Thus, we additionally fit both primary and secondary data together and compare our results with the primary-only fits to discern general trends between all best fits and to compare immune parameter differences between theorized higher and measured lower secondary immune response values.

In the absence of experimental measurements for many of our rate parameters, we use Monte Carlo parameter estimation runs to extensively explore the multi-dimensional parameter space of unknown parameter values. Of 33 parameters listed in Table 1, we fix seven parameters based on established values in the literature and vary the remaining 26, although some vary together. Tables 3 and 4 give more details. We establish ranges for each parameter, based on literature searches wherever possible, and keep unknown ranges as broad as reason and computation time allow. Using Latin hypercube sampling [58, 59], we segment each parameter’s range into 10 equal sections and randomly sample a value from each section. We simultaneously sample for all relevant parameters and randomly create a 26-tuple that contains one sampled value for every parameter. To explore a wide range of parameter value combinations, we repeat this 4,000 times. We then run a numerical simulation of the model for both primary and secondary infections to determine model dynamics and data-versus-model distances for each parameter tuple. We use least squares fitting to determine which parameter tuple best matches our clinical data. In the least squares analysis, we add over the sums of (ln(data) − ln(model))^2^ so that the higher order-of-magnitude antibody values do not mute ASC differences. We save the best-fit tuple and repeat this entire sampling process 25 times to establish a selection of many different parameter combinations that are optimal within their set. This allows us to identify parameter values that are relatively fixed across all best-fit tuples versus parameters that vary more widely without significantly affecting the goodness of fit (S1 Fig in the supporting information). We also look at partial rank correlation coefficients for all of our best-fit cases together to see which, if any, parameters are most crucial in fitting the model to primary and secondary infection data (Fig 3) [60, 61]. Finally, a grand Monte Carlo parameter estimation run chooses the single best-fit parameter tuple out of the 1,000,000 total sampled tuples for each study (EcSf2a-2 or 2457T) and each target (LPS or OMP) case.

**Table 3:** Best-Fit parameter values: Fit primary data only.

Parameter	Range and Units	Best-Fit	Best-Fit	Best-Fit	Best-Fit	Best-Fit	Best-Fit	Best-Fit	Best-Fit	Note
		EcSf2a-2	EcSf2a-2	2457T	2457T	EcSf2a-2	EcSf2a-2	2457T	2457T	
		LPS	LPS	LPS	LPS	OMP	OMP	OMP	OMP	
		Stable	Unstable	Stable	Unstable	Stable	Unstable	Stable	Unstable	
*σ*_*N*_	(0.01,1)/d	0.5173	0.6005	0.2067	0.3748	0.5173	0.6005	0.5641	0.383	
*σ*_*C*_	0.1/d	—	—	—	—	—	—	—	—	
*δ*_*SE*_	0.0001/d	—	—	—	—	—	—	—	—	
*δ*_*SI*_	0.0001/d	—	—	—	—	—	—	—	—	
*δ*_*SN*_	(0.1,1)/d	0.6655	0.4427	0.9858	0.9795	0.6655	0.4427	0.8182	0.853	
*δ*_*SC*_	0.0001/d	—	—	—	—	—	—	—	—	
*δ*_*AE*_	(0.01,0.5)/d	0.026	0.0264	0.1966	0.0524	0.026	0.0264	0.0493	0.026	
*δ*_*AI*_, *δ*_*G*_	(0.01,0.5)/d	0.308	0.4051	0.0259	0.0178	0.308	0.4051	0.01	0.057	(1)
*δ*_*PA*_	0.2/d	—	—	—	—	—	—	—	—	
*δ*_*PG*_	0.2/d	—	—	—	—	—	—	—	—	
*μ*_*E*_	(0.1,1)/d	0.1044	0.5856	0.8407	0.6888	0.1044	0.5856	0.7536	0.514	
*μ*_*I*_	(0.1,1)/d	0.4699	0.9248	0.8058	0.6685	0.4699	0.9248	0.5978	0.969	
*μ*_*NI*_, *μ*_*N*_	(0.5,0.9)/d	0.5891	0.4944	0.8956	0.8801	0.5891	0.4944	0.6258	0.557	(2)
*ω*	(0.01,1)/d	0.0227	0.0253	0.0806	0.1816	0.0227	0.0253	0.222	0.113	
*n*_*E*_, *n*_*I*_	(2E3,2E4) Ab/mL/cfu	67,67	9,955	3,298	9,036	6,767	9,955	3,025	13,986	(3)
*β*_*A*_	(5E4,5E7) (Ab/mL)/pc/d	122,111	213,061	844,742	271,928	122,111	213,061	4,532,685	6,229,820	
*β*_*G*_	(5E4,5E7) (Ab/mL)/pc/d	9,949,617	30,306,492	1,909,672	2,228,489	9,949,617	30,306,492	39,054,816	33,641,791	
*ϕ*_1_	(0.001,0.1)/d	0.0204	0.0902	0.0016	0.0681	0.0204	0.0902	0.002	0.073	
*ρ*	(0.001,0.1)/d	0.046	0.0021	0.0014	0.0067	0.046	0.0021	0.0298	0.003	
*ξ*_1_	(1, 1E4) pc/mc	5	2,697.9	179.3	3,303.4	5	2,697.9	3.2	3,144.8	
*ξ*_2_	(5E2,5E4) pc/mc	544	907	1,414	7,571	544	907	3,709	1,058	
*α*	(1E-16,1E-13)/(Ab/mL)/d	1.02E-15	1.95E-16	4.01E-16	3.38E-15	1.17E-15	1.95E-16	6.24E-14	2.40E-16	
*γ*	(1E-16,1E-13)/(Ab/mL)/d	7.01E-14	9.32E-14	1.10E-15	2.32E-15	7.01E-14	9.32E-14	2.60E-15	8.02E-14	
*ϕ*_2*A*_	(0.001,1)/cfu/d	0.0018	0.1816	0.0079	0.1796	0.0018	0.1816	0.0574	0.291	
*ϕ*_2*G*_	(0.001,1)/cfu/d	0.0238	0.0535	0.0017	0.0025	0.0238	0.0535	0.0013	0.019	
*k*_*A*_	(5E2,5E5) mc	33,146	65,773	21,611	46,902	33,146	65,773	974	527	
*k*_*G*_	(5E2,5E5) mc	259,192	4,192	10,536	504	259,192	4,192	2,786	2,084	
*λ*_*P*_	(1E-2,1E4) pc/cfu/d	0.01	0.0104	8.2818	0.1596	0.01	0.0104	0.0527	0.065	(4)
*λ*_*M*_	(1E-2,1E4) mc/cfu/d	0.01	0.0104	8.2818	0.1596	0.01	0.0104	0.0527	0.065	(4)
Υ	(0.05,1)	0.8932	0.9327	0.0636	0.3067	0.8932	0.9327	0.6436	0.067	
Delay	3.5 d	—	—	—	—	—	—	—	—	

Parameters values for the model are given along with their ranges and best-fit values using primary infection data only. The model is fit to primary infection data for the EcSf2a-2 trials or the 2457T study using LPS or OMP data separately. For each, the best-fit parameter set is given that produces a stable nontrivial equilibrium (wherein some immunity is established) versus an unstable nontrivial equilibrium (for which no immunity can establish). Parameters with fixed numbers are kept constant (–) while those with values were chosen by fitting from the range provided. Each column should be taken together as the best fit; every individual number is not necessarily the single best-fit value for that parameter. Notes: (1) Lumen Ab: *δ*_*AI*_ = *δ*_*G*_, (2) *μ*_*NI*_ = *μ*_*N*_, (3) *n*_*E*_ = *n*_*I*_, (4) All *λ* are set equal. Abbreviations: LPS: lipopolysaccharide, OMP: O-membrane proteins

**Table 4:** Best-Fit parameter values: Fit primary and secondary data.

Parameter	Range and Units	Best-Fit	Best-Fit	Best-Fit	Best-Fit	Best-Fit	Notes
		EcSf2a-2	2457T	2457T	EcSf2a-2	2457T	
		LPS	LPS	LPS	OMP	OMP	
		Stable	Stable	Unstable	Stable	Unstable	
*σ*_*N*_	(0.01,1)/d	0.8024	0.7524	0.3748	0.6253	0.3826	
*σ*_*C*_	0.1/d	—	—	—	—	—	
*δ*_*SE*_	0.0001/d	—	—	—	—	—	
*δ*_*SI*_	0.0001/d	—	—	—	—	—	
*δ*_*SN*_	(0.1,1)/d	0.7416	0.7275	0.9795	0.7436	0.8527	
*δ*_*SC*_	0.0001/d	—	—	—	—	—	
*δ*_*AE*_	(0.01,0.5)/d	0.0479	0.0668	0.0524	0.4125	0.0263	
*δ*_*AI*_, *δ*_*G*_	(0.01,0.5)/d	0.4969	0.0225	0.0178	0.1273	0.0569	(1)
*δ*_*PA*_	0.2/d	—	—	—	—	—	
*δ*_*PG*_	0.2/d	—	—	—	—	—	
*μ*_*E*_	(0.1,1)/d	0.8481	0.9696	0.6888	0.1319	0.5135	
*μ*_*I*_	(0.1,1)/d	0.9218	0.6723	0.6685	0.9138	0.9685	
*μ*_*NI*_, *μ*_*N*_	(0.5,0.9)/d	0.6455	0.5144	0.8801	0.4049	0.5573	(2)
*ω*	(0.01,1)/d	0.0204	0.102	0.1816	0.0213	0.1135	
*n*_*E*_, *n*_*I*_	(2E3,2E4) Ab/mL/cfu	9,191	13,637	9,036	14,259	13,986	(3)
*β*_*A*_	(5E4,5E7) (Ab/mL)/pc/d	119,091	410,585	271,928	2,713,011	6,229,820	
*β*_*G*_	(5E4,5E7) (Ab/mL)/pc/d	36,708,315	971,487	2,228,489	22,509,577	33,641,791	
*ϕ*_1_	(0.001,0.1)/d	0.0251	0.0067	0.0681	0.0041	0.073	
*ρ*	(0.001,0.1)/d	0.0986	0.0056	0.0067	0.0273	0.0031	
*ξ*_1_	(1, 1E4) pc/mc	61.3	1.8	3,303	10.2	3,144	
*ξ*_2_	(5E2,5E4) pc/mc	664	4,882	7,571	768	1,058	
*α*	(1E-16,1E-13)/(Ab/mL)/d	2:69E-16	7.96E-15	3.38E-15	1.40E-15	2.40E-16	
*γ*	(1E-16,1E-13)/(Ab/mL)/d	9.44E-14	1.12E-16	2.32E-15	7.68E-14	8.02E-14	
*ϕ*_2*A*_	(0.001,1)/cfu/d	0.8159	0.0135	0.1796	0.0034	0.2909	
*ϕ*_2*G*_	(0.001,1)/cfu/d	0.1987	0.0013	0.0025	0.1283	0.0191	
*k*_*A*_	(5E2,5E5) mc	110,919	1,263	46,902	19,136	527	
*k*_*G*_	(5E2,5E5) mc	551	1,222	504	13,904	2,084	
*λ*_*P*_	(1E-2,1E4) pc/cfu/d	0.0165	0.4733	0.1596	0.0116	0.0649	(4)
*λ*_*M*_	(1E-2,1E4) mc/cfu/d	0.0165	0.4733	0.1596	0.0116	0.0649	(4)
Υ	(0.05,1)	0.7997	0.7898	0.3067	0.6309	0.0667	
Delay	3.5 d	—	—	—	—	—	

Parameters values for the model are given along with their ranges and best-fit values using primary and secondary infection data. The model is fit jointly to both primary and secondary infection data for the EcSf2a-2 trials or the 2457T study using LPS or OMP data separately. For each, the best-fit parameter set is given that produces a stable nontrivial equilibrium (wherein some immunity is established) versus an unstable nontrivial equilibrium (for which no immunity can establish). No parameter set was found that produced a stable nontrivial equilibrium for 2457T OMP data or an unstable nontrivial equilibrium for EcSf2a-2 LPS or OMP data when both primary and secondary infection data are fit. Parameters with fixed numbers are kept constant (–) while those with values were chosen by fitting from the range provided. Each column should be taken together as the best fit; every individual number is not necessarily the single best-fit value for that parameter. Notes: (1) Lumen Ab: *δ*_*AI*_ = *δ*_*G*_, (2) *μ*_*NI*_ = *μ*_*N*_, (3) *n*_*E*_ = *n*_*I*_, (4) All *λ* are set equal.

One pattern we notice is that the best 25 fits in most grand runs generate some best-fit parameter tuples that correspond with a stable positive nontrivial immune equilibrium and others that instead have only the trivial equilibrium where immunity is not lasting. This matches the analytical observation that there is a transcritical bifurcation at which the nontrivial equilibrium loses stability. To compare the parameter values and model results associated with a stable versus unstable nontrivial equilibrium, we isolate the best fitting stable nontrivial equilibrium run and the best unstable nontrivial equilibrium run, in which only the trivial equilibrium is biologically relevant and stable. This is done for each bacterial target and for each clinical study. The grand best-fit parameter tuples in each case are given in Tables 3 and 4, depending upon if the model is fit to primary infection data only or to both primary and secondary infection data, respectively. Thus, this parameter fitting process gives us a wealth of outputs to parse for identifying the key biological rates and interactions responsible for conferring or failing to confer protective immunity against *Shigella*.

We use GenSSI to evaluate which parameters are structurally identifiable versus which are difficult to uniquely determine [62]. If all state variables are measurable and if the system launches from a nonimmune state (i.e., the trivial equilibrium), GenSSI finds that *δ*_*SE*_, *δ*_*SI*_, *μ*_*E*_, *μ*_*I*_, *μ*_*NI*_, *μ*_*N*_, *ω*, *β*_*A*_, *β*_*G*_, *α*, *γ*, *λ*_*P*_, and *λ*_*M*_ are globally structurally identifiable. No parameters were found to be structurally non-identifiable. However, the identifiability of parameters not listed here could not be resolved due to computational limitations in calculating sufficient Lie derivatives to parse underlying relationships. If we consider only those state variables for which we have data to be measurable, then using 12 Lie derivatives in GenSSI only definitively determines that *σ*_*C*_ and *δ*_*SC*_ (parameters governing *Shigella* dynamics inside of epithelial cells) are structurally non-identifiable. Lack of identifiability is likely for more parameters in this case due to our inability to measure bacterial levels internally in humans.

We conduct numerical sensitivity analysis to discern the effects of individual parameters upon the severity of the *Shigella* infection, as measured by the number of bacteria within the epithelium, *S*_*C*_, on day 7 after infection. Every parameter is varied one at a time from the model parameterized by the overall best-fit parameter tuple. This is done using as baseline the best-fit tuple for a stable nontrivial equilibrium as well as separately the best-fit tuple for an unstable nontrivial equilibrium, and results are compared when the model fits primary data only versus fits both primary and secondary data. Furthermore, we carry out this sensitivity analysis for each study (EcSf2a-2 or 2457T) and for each bacterial target (LPS or OMP). We compare general trends across all of these scenarios in order to discern which model parameters are key to establishing or preventing severe epithelial infections across all examined biological parameterizations. Parameters are varied at minimum across the ranges given in Table 1 and sometimes across wider ranges. Similar parameters are varied together. The four parameter groups that we identify as sensitive through this analysis under our strictest protection threshold are varied across the following ranges: (0.01, 1)/d for *μ*_*E*_ and *μ*_*I*_; (10^0^, 10^6^) ASC/B_M_ on a log scale for *ξ*_1_ and *ξ*_2_; (10^2^, 10^10^) Ab/mL/ASC/d on a log scale for *β*_*A*_ and *β*_*G*_; and (10^2^, 10^8^) B_M_ on a log scale for *κ*_*A*_ and *κ*_*G*_.

The goal of our sensitivity analysis is to identify individual parameters that, when altered, have the capacity to move the resulting dynamics below a protection threshold. Key parameters that do so are potential immune correlates of protection for *Shigella* and are highlighted for continued investigation in the future. However, it is not known what degree of epithelial invasion by *Shigella* must be prevented for the host to remain unsymptomatic [3, 35]. When estimating this threshold for our model, it should be kept in mind that our model does not incorporate cell-mediated immunity (e.g., T-cells) and thus assumes all bacteria that infect the epithelium escape immune action; *in vivo* conditions would likely include a naive CMI response that could handle a larger number of bacteria invading the epithelium than the model would suggest. Thus, the protective threshold desired for modeling purposes is the *effective* number of bacteria *in vivo* that can not only infect the epithelium by day 7 but also escape all immune action without the host becoming symptomatic. In lieu of a known protective threshold, we examine immune requirements to keep the number of *Shigella* in the epithelium below either 10 cfu or 100 cfu by day 7 of infection. (Nevertheless, a model threshold of 10 cfu translates to a higher actual number of allowed epithelially invasive bacteria if CMI is present.) Whether immunity that keeps epithelial bacteria below 10 or 100 cfu is protective is not known. Importantly, we will see in our results that the baseline best-fit parameter tuples do not sufficiently prevent the epithelial infection in any case under any parameterization as judged by the 10 cfu threshold. Under the 100 cfu threshold, results will show that three cases would be deemed protective on average with their best-fit parameterizations: (1) fitting to EcSf2a-2 LPS primary immunity data with a stable nontrivial equilibrium, (2) fitting to EcSf2a-2 OMP primary immunity data with a stable nontrivial equilibrium, and (3) fitting to EcSf2a-2 OMP primary and secondary immunity data with a stable nontrivial equilibrium. Since the EcSf2a-2 vaccine demonstrated 27-36% efficacy, it was considered insufficiently protective at the group and population level. This partial efficacy informs the modeling threshold we use as a surrogate for predicting clinical outcome. Since under a 100 cfu threshold the model would have demonstrated complete protection in the three aforementioned cases and since such protection was not demonstrated clinically, we use a 10 cfu threshold as our target level for protection. Nevertheless, we include information using both bacterial thresholds to elucidate how modeling results are impacted by threshold choice.

## Results

### Primary infection data fits

**Fig 4:**
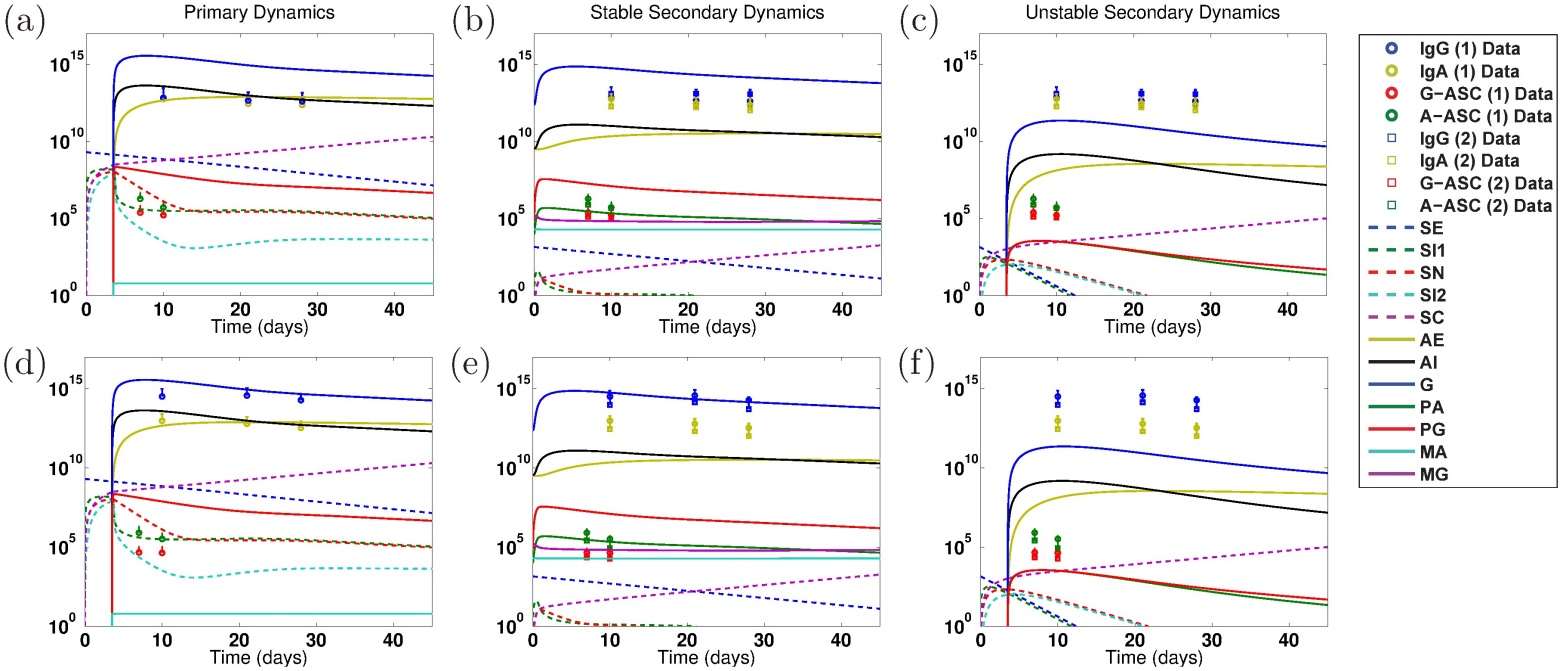
Numerical simulations of model dynamics for the best fits to primary EcSf2a-2 data (ET: 2457Tx1). Vertical axis units are number of Ab per mL (highest three lines: *A*_*E*_, *A*_*I*_, *G*), number of ASC (middle lines: *P*_*A*_, *P*_*G*_), number of B_M_ (lower flat lines: *M*_*A*_, *M*_*G*_), and bacterial cfu (lower dashed lines: *S*_*E*_, *S*_*I*1_, *S*_*N*_, *S*_*I*2_; rising purple dashed line: *S*_*C*_). The model is fit to LPS (top row: a–c) or OMP (lower row: d–f) primary infection data from control volunteers who received no vaccine and a wild-type challenge in the EcSf2a-2 vaccine trials (ET: 2457Tx1). The model is fit only to primary (1) infection data (ET: 2457Tx1). Secondary (2) infection data (ET: EcSf2a-2x1, 2457Tx1) from EcSf2a-2 volunteers who received both the vaccine and subsequent challenge are given for reference but were not used for these fits. Primary infection dynamics are shown for the stable nontrivial equilibrium’s best-fit parameters in (a) and (d). The secondary infection dynamics predicted under the best primary infection parameter fit that corresponds with a stable nontrivial equilibrium (i.e., some immunity) are given in (b) and (e), whereas dynamics from the best primary fit that results in an unstable nontrivial equilibrium are given in (c) and (f). To match clinical conditions, the primary infection is initialized at 2 × 10^9^ cfu of luminal *Shigella*, whereas secondary dynamics are initiated at 1,400 cfu. Legend abbreviations match model variable names, without subscripts. *S*: *Shigella*, *A*: IgA, *G*: IgG, *M*: B_M_, *P*: ASC, *I*: in Lamina Propria, *E*: Luminal, *C*: Epithelial, *N* Engulfed.

**Fig 5:**
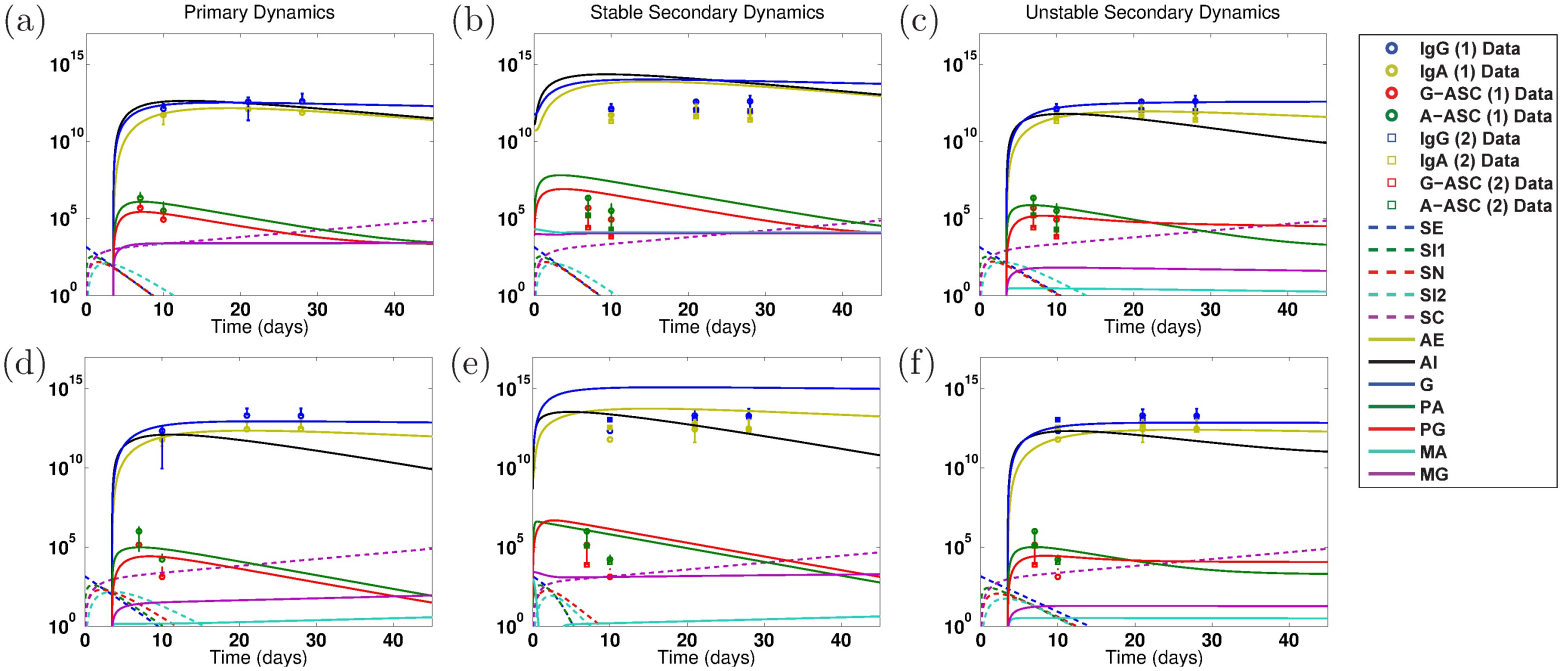
Numerical simulations of model dynamics for the best fits to primary 2457T rechallenge data (2T: 2457Tx1). Vertical axis units are number of Ab per mL (highest three lines: *A*_*E*_, *A*_*I*_, *G*), number of ASC (middle lines: *P*_*A*_, *P*_*G*_), number of B_M_ (lower flat lines: *M*_*A*_, *M*_*G*_), and bacterial cfu (lower dashed lines: *S*_*E*_, *S*_*I*1_, *S*_*N*_, *S*_*I*2_; rising purple dashed lines: *S*_*C*_). The model is fit to LPS (a–c) or OMP (d–f) primary infection data from volunteers who received a single wild-type challenge in the 2457T rechallenge study (2T: 2457Tx1). The model is only fit to primary (1) infection data (2T: 2457Tx1). Secondary (2) infection data (2T: 2457Tx2) from 2457T rechallenge study volunteers who received two wild-type challenges (and no previous vaccine) are given for reference but were not used for these fits. No tertiary infection data (2T: EcSf2a-2x1, 2457Tx2) are included. To match clinical conditions, both the primary and secondary infections are initialized at 1,400 cfu of luminal *Shigella*. Abbreviations and distinctions between Primary (a, d), Stable Secondary (b, e), and Unstable Secondary (c, f) Dynamics are as described in the previous figure caption.

When we parameterize the model with primary infection data from the vaccine trials (ET: 2457Tx1) or rechallenge study (2T: 2457Tx1), we observe decent fits of the model to primary data, as shown in Panels a and d of Figs 4 and 5 for the EcSf2a-2 vaccine and 2457T rechallenge studies, respectively. This is expected as there are enough unknown parameters that our model has a high likelihood of fitting the data. Some parameter values can range broadly without substantially affecting fits, while other best-fit values cluster together within wider ranges. As seen in S1 Fig, clustered parameter values determine equilibrium stability (*σ*_*N*_ and *δ*_*SN*_), affect bacterial infectivity and Ab migration (*μ*_*E*_, *μ*_*I*_, and *ω*), and control new B-cell formation (*λ*_*P*_ and *λ*_*M*_, which fall broadly in the lower half of their range). We further examine which parameters most affect the model’s fit to data by calculating partial rank correlation coefficients (PRCC), which are plotted in Fig 3. When we use PRCC to compare the top 25 best-fit tuples for each case across all cases, no parameter has a PRCC value beyond ±0.5, although some parameters (*μ*_*I*_, *ξ*_2_, *ϕ*_1_, *ϕ*_2*A*_, *ϕ*_2*G*_) have a p-value smaller than 0.05. Thus, PRCC analysis indicates that no parameters are the primary contributors to the goodness of fit of the parameterized model to data; a combination of parameters must be responsible for the fits. It is important to note that PRCC analysis describes which parameters are responsible for the model fitting to data, not which parameters are responsible for reducing the epithelial infection or host symptoms; we explore the latter, a key goal of vaccination, with numerical sensitivity analysis.

Our best-fit parameter values when fitting the model to primary infection data are given in Table 3. Given our model’s many fitted parameter values, we avoid pitfalls of overfitting by never considering our best-fit parameters to each individually be the best possible parameter value for matching the model to data; rather, we compare commonalities across many best-fit parameter tuples to observe general trends and conclusions that can be made, regardless of whether each parameter value is itself optimized. In this way, we ensure that our conclusions are robust across an array of feasible parameterizations.

We first fit the model to the primary EcSf2a-2 vaccine trial data (ET: 2457Tx1) and separately to the primary 2457T rechallenge data (2T: 2457Tx1). Specifically, we fit the model simultaneously to the serum IgA, serum IgG, A-ASC, and G-ASC data targeting LPS for each study. We separately fit the model to the same data targeting OMP for each study. The fits to primary infection data are plotted in Panels a and d of Figs 4 (EcSf2a-2) and 5 (2457T). We then use the model parameterized against each primary data set to numerically predict bacterial and immune dynamics during a subsequent *Shigella* infection. The Latin hypercube random samples as a whole were different for all cases, but some of the 1,000,000 tuples generated by Matlab’s LHS solver overlapped in parameter values with other cases; surprisingly, the same parameter tuple was optimal for both EcSf2a-2 LPS data and EcSf2a-2 OMP data when fitting the model separately to these primary infection data (but not when fitting to both primary and secondary infection data). This remained true after rerunning these simulations with new random seeds, and thus LPS and OMP EcSf2a-2 primary (but not secondary) data fits have identical characteristics in our results.

The disease-free IgA/IgG nontrivial equilibrium values given in Table 2 are subject to stability conditions, and we analytically and numerically examine whether each best-fit parameterization produces a stable or unstable disease-free state. Model predictions based on fits to primary infection data with predicted secondary infection dynamics are plotted in Panels b, c, e, and f in Figs 4 (EcSf2a-2) and 5 (2457T) and overlaid for comparison (but not fit) with clinical data of secondary immune levels from the two studies. Secondary infection data from the EcSf2a-2 trials show measurements taken after challenging volunteers with wild-type *Shigella* after the candidate vaccine had already been administered (ET: EcSf2a-2x1, 2457Tx1); data from the 2457T rechallenge study come from six rechallenge patients who received a sequential double wild-type challenge (2T: 2457Tx2). The EcSf2a-2 studies resulted in 27-36% efficacy at establishing immunity, while five of the six subjects in the 2457T study did not get ill upon rechallenge [32, 34].

Results with a stable nontrivial equilibrium when the model is fit only to primary infection data are shown in comparison with measured data values in Panels b and e in Figs 4 (EcSf2a-2) and 5 (2457T). In all cases, the data (taken from Fig 1) have a not-significant difference, and in some cases a decrease, in antibody and immune levels from a primary to a secondary infection. This lack of rise in measured immunity is discordant with the model’s predictions in Panel b in Fig 4 and Panels b and e in Fig 5 of heightened secondary immunity arising from a stable nontrivial equilibrium. That is, when a stable nontrivial equilibrium (and hence some immunity) is established by a primary infection, the model overestimates the antibody and ASC levels during a secondary infection in comparison with the data by predicting an increase of roughly 1–3 orders of magnitude in secondary antibody and ASC numbers in all cases except for the EcSf2a-2 OMP response (Panel e in Fig 4). This is consistent with published literature that describes a blunted ASC response to challenge in blood among subjects who are protected from *Shigella*, presumed to be due to a secondary immune response at the mucosa (as the model predicts) [32]. Interestingly, every grand Monte Carlo fitting round with fits to primary infection data produced a few best-fit parameter tuples that corresponded with an unstable nontrivial equilibrium in addition to those that produced a stable nontrivial equilibrium. Thus, primary infection data can be fit both with parameter combinations that do not lead to an immune state and others that do lead to some immunity. If we look at the parameterizations that produce unstable nontrivial equilibria and initialize our model from the trivial equilibrium (which assumes no lasting immunity from the previous infection), then our model matches the clinical secondary infection data remarkably well for the 2457T data (Panels c and f in Fig 5). This is perhaps unsurprising because the primary and secondary infection data are similar, and the model was fit to the primary data. Nevertheless, Fig 5 reveals that, on average, wt anti-LPS and anti-OMP immune levels during a secondary wt challenge (2T: 2457Tx2) are mathematically nearly identical to initiating an infection from a trivial, non-immune state. That is, the model best matches wt challenge data if no lasting LPS or OMP immunity is established.

**Fig 6:**
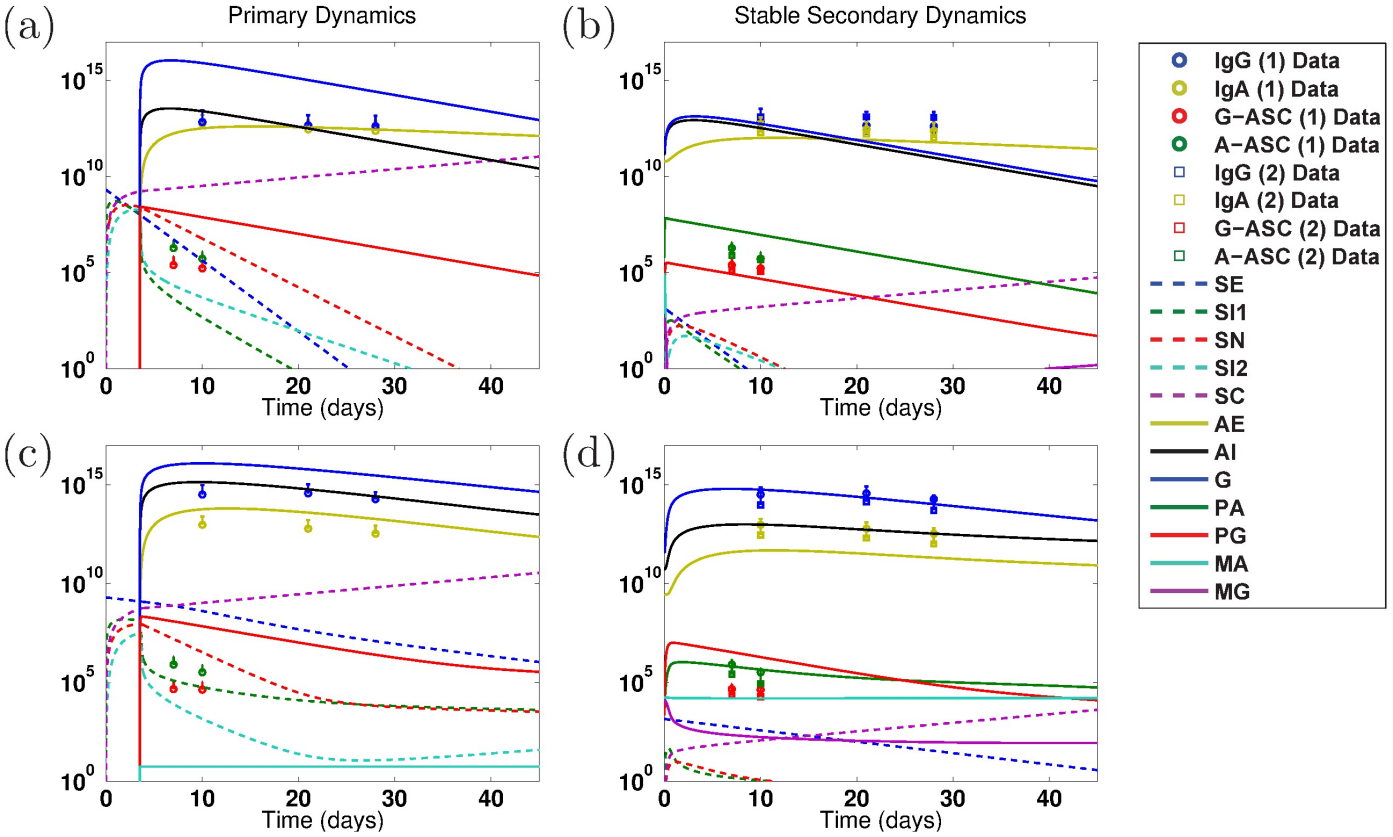
Numerical simulations of model dynamics for the best fits using both primary and secondary EcSf2a-2 data (ET: 2457Tx1 and ET: EcSf2a-2x1, 2457Tx1, respectively). Vertical axis units are number of Ab per mL (highest three lines: *A*_*E*_, *A*_*I*_, *G*), number of ASC (middle lines: *P*_*A*_, *P*_*G*_), number of B_M_ (lower flat lines: *M*_*A*_, *M*_*G*_), and bacterial cfu (lower dashed lines: *S*_*E*_, *S*_*I*1_, *S*_*N*_, *S*_*I*2_; rising purple dashed line: *S*_*C*_). The model parameters are fit to both primary and secondary infection data for LPS (a–b) or OMP (c–d). Primary (1) infection data is from control volunteers who received no vaccine and a wild-type challenge in the EcSf2a-2 vaccine trials (ET: 2457Tx1). Secondary (2) infection data is from volunteers who received both the EcSf2a-2 vaccine and subsequent wild-type challenge (ET: EcSf2a-2x1, 2457Tx1). To match clinical conditions, the primary infection is initialized at 2 × 10^9^ cfu of luminal *Shigella*, whereas secondary dynamics are initiated at 1,400 cfu. No best-fit parameterizations gave an unstable nontrivial equilibrium when fitting both primary and secondary infection EcSf2a-2 data. Resulting model dynamics using the joint fits show predicted Primary (a, c) and Stable Secondary (b, d) Dynamics as described for previous figures.

**Fig 7:**
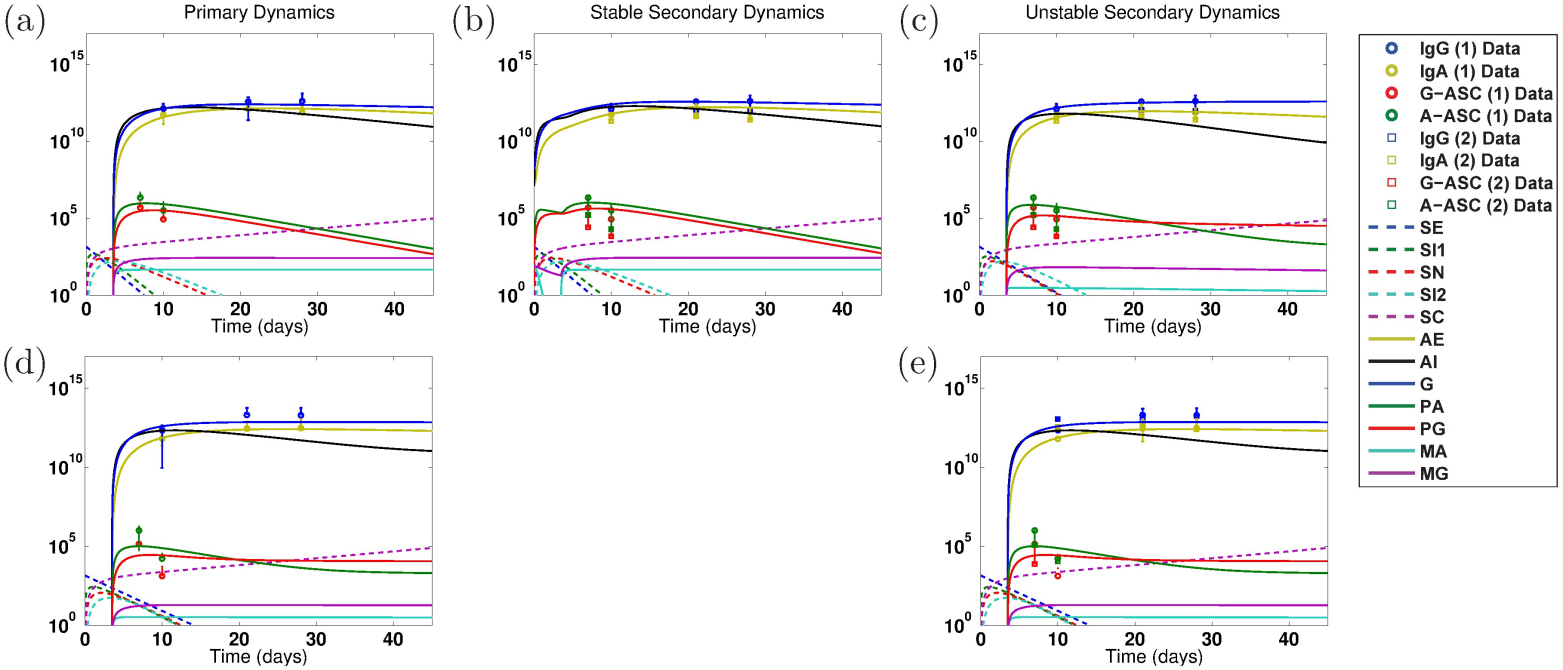
Numerical simulations of model dynamics for the best fits using both primary and secondary 2457T rechallenge data (2T: 2457Tx1 and 2T: 2457Tx2, respectively). Vertical axis units are number of Ab per mL (highest three lines: *A*_*E*_, *A*_*I*_, *G*), number of ASC (middle lines: *P*_*A*_, *P*_*G*_), number of B_M_ (lower flat lines: *M*_*A*_, *M*_*G*_), and bacterial cfu (lower dashed lines: *S*_*E*_, *S*_*I*1_, *S*_*N*_, *S*_*I*2_; rising purple dashed line: *S*_*C*_). The model parameters are fit to both primary and secondary infection data for LPS (a–c) or OMP (d–e). Primary (1) infection data is from volunteers who received a single wild-type challenge in the 2457T rechallenge study (2T: 2457Tx1). Secondary (2) infection data is from volunteers who received two 2457T challenges (one as controls during the EcSf2a-2 trial and a second one during the 2457T rechallenge study, 2T: 2457Tx2). No tertiary vaccine-challenge-challenge infection data are included (2T: EcSf2a-2x1, 2457Tx2). To match clinical conditions, both the primary and secondary infections are initialized at 1,400 cfu. No best-fit parameterizations gave a stable nontrivial equilibrium when the model was fit to both primary and secondary 2457T OMP data. Resulting model dynamics using the joint fits show predicted Primary (a, d), Stable Secondary (b), and Unstable Secondary (c,e) Dynamics as described in previous figures.

The predicted secondary infection levels following a stable disease-free state match data in the case of EcSf2a-2 OMP, which suggests that some anti-OMP immunity (not necessarily protective) was established by the EcSf2a-2 vaccine. Meanwhile, our model predictions fail to match measured secondary infection data for the EcSf2a-2 trials with a stable or unstable equilibrium (that is, with or without some immunity establishing) in every case except when OMP immunity is established (Panel e in Fig 4 versus Panels b, c, and f in Fig 4). The explanation is likely that, although the primary and secondary data are similar, the initial infection dose varies widely in the EcSf2a-2 trials: 2 × 10^9^ cfu for a primary vaccine dose versus 1,400 cfu for a secondary challenge dose. Thus, the model did not replicate vaccine trial data when fit solely to primary infection dynamics except when investigating OMP immune responses. We next ask whether we can obtain better information about the EcSf2a-2 vaccine trial by fitting both primary and secondary infection data, and we investigate whether our 2457T and EcSf2a-2 OMP modeling results are robust across other parameterizations. However, our primary data fits inform our primary and secondary data fit results and indicate, for instance, why no best fit parameterizations are found for certain cases (unstable and stable secondary dynamics, respectively) in Figs 6 and 7.

### Primary and secondary infection data fits

To see if model results are robust or are artifacts of our parameterization process, we redo the data fits but simultaneously fit both the primary and secondary infection data. We wish to discern if stable nontrivial equilibria, which correspond to establishing some level of immunity (although possibly not protective at the group level), are possible with our model while fitting data other than just EcSf2a-2 OMP, and we seek to corroborate the primary-fit EcSf2a-2 OMP and the primary-fit 2457T modeling results. Thus now in addition to primary infection data (ET: 2457Tx1), we use the data from volunteers who received both the EcSf2a-2 vaccine and a wild-type 2457T *Shigella* challenge as the secondary infection data (ET: EcSf2a-2x1, 2457Tx1) in the EcSf2a-2 trial fits. For the 2457T secondary infection fits, we match primary infection data (2T: 2457Tx1) as well as data from volunteers who were controls in the EcSf2a-2 trial (and thus were challenged but did not receive the vaccine candidate) and then were rechallenged with wild-type *Shigella* during the 2457T rechallenge study (2T: 2457Tx2). The results are plotted in Figs 6 (EcSf2a-2) and 7 (2457T) and predicted antibody, ASC, and B_M_ equilibrium numbers are given in Table 2. Best-fit parameter values are listed in Table 4. We again separately examine best-fit parameterizations that produce a stable versus unstable nontrivial equilibrium.

The fits for the OMP measurements from the primary and secondary 2457T study generated only best-fit parameters that corresponded with an unstable nontrivial equilibrium; no stable best fits were found. For every other case, we produce a best-fit parameterization with a stable nontrivial equilibrium that matches both the primary and secondary infection data. In fact, for the EcSf2a-2 trial data (Fig 6), we found no best-fit parameterizations that produced an unstable nontrivial equilibrium and could mimic both primary vaccine data (initialized at 2 × 10^9^ cfu) and secondary challenge data (with a 1,400 cfu infection dose). That is, of 25 grand best-fit parameterizations for EcSf2a-2 LPS data and separately for EcSf2a-2 OMP data, all produced only a stable nontrivial immunity. This strongly suggests that the EcSf2a-2 vaccine established some lasting anti-LPS and anti-OMP humoral immunity, although perhaps not at levels that are protective for the population.

Furthermore, we examine the number of *Shigella* that have invaded the epithelium by day 7 of infection as a proxy for disease severity. This is the day 7 value of the rising purple dashed *S*_*C*_ curve in Figs 4–7. Day 7 epithelial *Shigella* levels are listed in Table 5 for all cases. We note that while all cases have epithelial infections above the 10 cfu threshold, three cases have values below the weaker 100 cfu threshold. These three are (1) fitting to EcSf2a-2 LPS primary infection data with a stable nontrivial equilibrium, (2) fitting to EcSf2a-2 OMP primary infection data with a stable nontrivial equilibrium, and (3) fitting to EcSf2a-2 OMP primary and secondary infection data with a stable nontrivial equilibrium. This too suggests that the EcSf2a-2 vaccine was effective at preventing some disease and establishing some immunity, which is corroborated by the 27-36% vaccine efficacy. However, the level of immunity created by the EcSf2a-2 vaccine was not sufficiently efficacious at a group level. This indicates that even 100 cfu of bacteria (in the absence of a CMI response) invading the epithelium by day 7 are predicted to induce symptoms in the host, so the model’s threshold for protection must be lower than 100 cfu of epithelial bacteria. Hence, we use 10 cfu as our target threshold.

**Table 5:** Epithelial *Shigella* levels: Day 7 of secondary infection.

Study & Target	F1U (cfu)	F12U (cfu)	F1S (cfu)	F12S (cfu)
EcSf2a-2 LPS	2,089	—	36	1,236
2457T LPS	1,581	1,581	1,656	1,996
EcSf2a-2 OMP	2,089	—	36	85
2457T OMP	1,757	1,757	1,044	—

The number of bacteria (cfu) in the epithelium (*S*_*C*_ in the model) on day 7 of a secondary infection is given for every scenario investigated. Hosts with epithelial infection numbers above 100 cfu are likely symptomatic. Hosts with between 10 and 100 cfu might be symptomatic. No cases had less than 10 cfu of epithelial bacteria on day 7. The cases considered are as follows. Rows are data studies (EcSf2a-2 or 2457T) and bacterial targets (LPS or OMP) in the same order as in sensitivity analysis figures and supplementary figures. Columns are day 7 secondary infection levels for the following parameterizations (left to right): fit primary infection data with an unstable nontrivial equilibrium (F1U), fit primary and secondary infection data with an unstable nontrivial equilibrium (F12U), fit primary infection data with a stable nontrivial equilibrium (F1S), and fit primary and secondary infection data with a stable nontrivial equilibrium (F12S). F1U, F1S, and F12S (Columns 1, 3, and 4) correspond with baseline levels (black horizontal line) in the upcoming sensitivity analysis figures. In cases marked —, no parameterizations were found.

Turning to the wild-type 2457T results (Fig 7), the situation is different. When we fit the model to both primary and secondary infection data for a wt challenge, we see that both stable and unstable nontrivial equilibrium cases fit the LPS data well, while only an unstable equilibrium fits OMP data. Furthermore, the nontrivial equilibrium values in Table 2 indicate that the 2457T LPS stable best-fit parameterization (for fitting primary and secondary infection data) establishes negligibly small numbers of Ab, ASC, or B_M_. Thus, the model only fits the 2457T rechallenge data when little-to-no lasting immunity is established. This is further supported by the day 7 epithelial infection levels (Table 5), which are much larger than 100 cfu for all wt 2457T cases. This would suggest a heavily symptomatic host.

In fact, in all best-fit parameterizations of our model using either EcSf2a-2 or 2457T primary or secondary infection data, bacterial numbers inside of the epithelium rise quickly and uncontrollably. This can be seen by the rising purple dashed *S*_*C*_ curves in every panel of Figs 4–7 and by the day 7 levels in Table 5. In three EcSf2a-2 cases previously mentioned, these day 7 epithelial levels are kept below 100 cfu, and yet the vaccine was still not sufficiently efficacious at a group level. In no case is the day 7 epithelial *Shigella* level kept below the lower threshold of 10 cfu by the humoral immune response. While the true threshold for an asymptomatic host is unknown, these results together indicate that *Shigella* bacteria are not sufficiently prevented from invading the epithelium by anti-LPS or -OMP immune responses in any investigated scenario or study. Thus, the model predicts epithelial escape of the bacteria (and a symptomatic host) in all best-fit cases regardless of parameterization. That is, in 1,000,000 Monte Carlo runs and 25 best-fit parameter tuples observed for each case, not one predicted that an anti-LPS or anti-OMP vaccine would prevent an epithelial *Shigella* infection below a 10 cfu threshold, and the three cases that kept the epithelial infection below 100 cfu are known not to have been protective on average at a group level. Thus, our modeling results indicate that humoral immunity against only LPS and OMP, regardless of whether established via vaccine or wild-type infection, will not be protective against *Shigella*. Below, we amend this slightly while exploring the parameter space further with sensitivity analysis to determine whether altering individual immune response rates can increase the effectiveness of an LPS- or OMP-targeted vaccine.

### Sensitivity analysis

**Fig 8:**
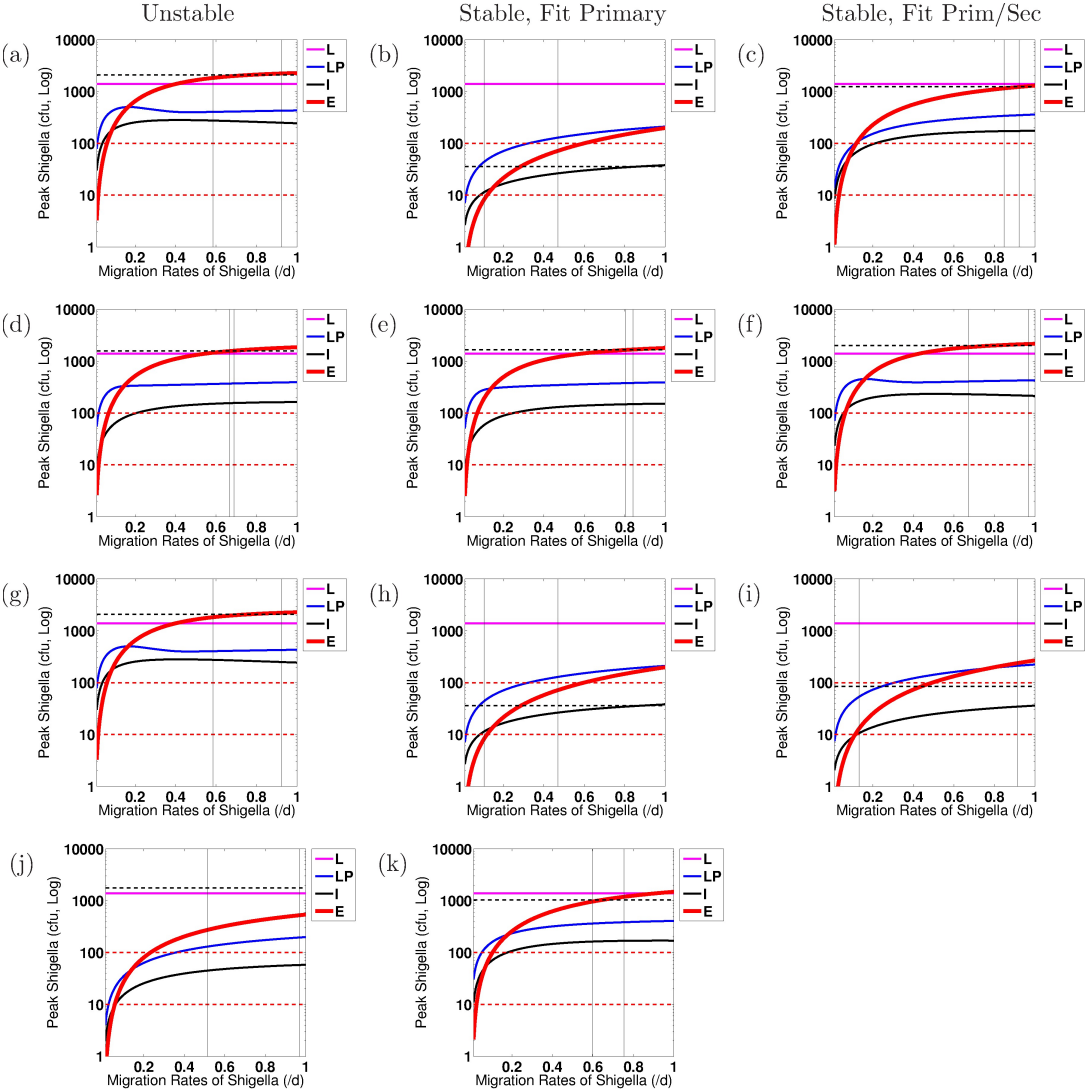
Predicted peak *Shigella* cfu levels when parameters *μ*_*E*_ and *μ*_*I*_ are varied together from 0.01 to 1/d. These control the rate at which *Shigella* migrates from the lumen into the lamina propria (LP) or from the LP into the epithelium. All other parameters are fixed to the best-fit parameter values given in Tables 3 and 4 for the model when fit to (a–c) EcSf2a-2 trials LPS data, (d–f) 2457T study LPS data, (g-i) EcSf2a-2 trials OMP data, or (j–k) 2457T study OMP data. Model predictions for peak *Shigella* values by day 7 of infection are shown for the lumen (L), LP, engulfed inside innate immune cells (I), or in the epithelium (E). These are primary (a,d,g,j) and positive, stable secondary (b,e,h,k) infection dynamics fit to primary infection data only or are positive, stable secondary infection dynamics (c,f,i) resulting from fitting both primary and secondary infection data. Results with an unstable nontrivial equilibrium produced when fitting to both primary and secondary infection data are not shown due to strong similarity to the displayed unstable primary infection best-fit results. No best-fit parameter set for OMP measurements from the 2457T rechallenge study produced a positive, stable nontrivial equilibrium when fitting both primary and secondary infection data. Vertical lines indicate best-fit parameter values. The horizontal black line shows the best-fit day 7 epithelial *Shigella* infection numbers for comparison to the thick red E curve. The red horizontal lines show 10 cfu and 100 cfu thresholds for assessing protection from epithelial infection. Corresponding peak immune numbers are given in S2 Fig.

**Fig 9:**
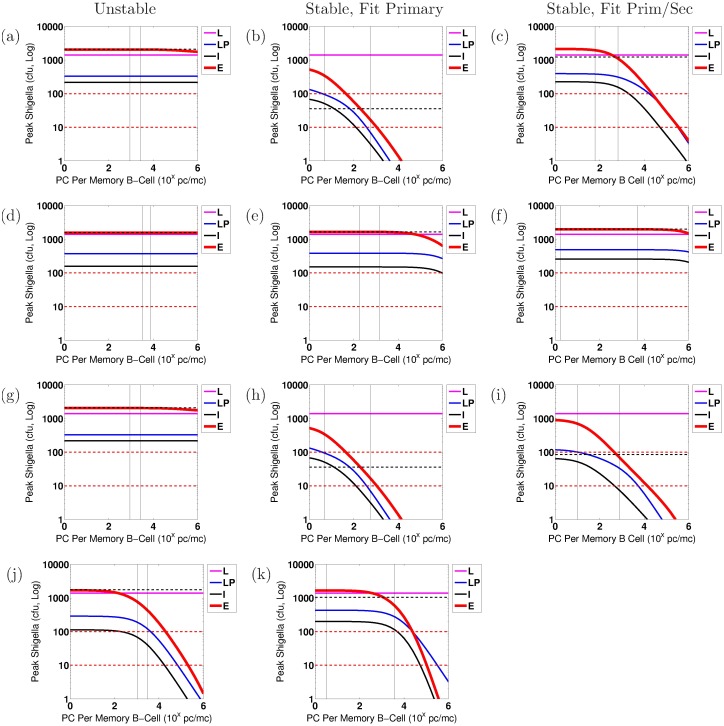
Predicted peak *Shigella* cfu levels when parameters *ξ*_1_ and *ξ*_2_ are varied together on a log scale from 10^0^ to 10^6^ ASC/B_M_. These control the rates at which new ASC are generated by B_M_ in the absence versus presence of antigenic stimulation, respectively. All other parameters are fixed to the best-fit parameter values given in Tables 3 and 4 for the model when fit to (a–c) EcSf2a-2 trials LPS data, (d–f) 2457T study LPS data, (g-i) EcSf2a-2 trials OMP data, (j–k) 2457T study OMP data. Model predictions for peak *Shigella* values by day 7 of infection are shown for the lumen (L), lamina propria (LP), engulfed inside innate immune cells (I), or in the epithelium (E). These are primary (a,d,g,j) and positive, stable secondary (b,e,h,k) infection dynamics fit to primary infection data only or are positive, stable secondary infection dynamics (c,f,i) resulting from fitting both primary and secondary infection data. Results with an unstable nontrivial equilibrium produced when fitting to both primary and secondary infection data are not shown due to strong similarity to the displayed unstable primary infection best-fit results. No best-fit parameter set for OMP measurements from the 2457T rechallenge study produced a positive stable nontrivial equilibrium when fitting both primary and secondary infection data. Vertical lines indicate best-fit parameter values. The horizontal black line shows the best-fit day 7 epithelial *Shigella* infection numbers for comparison to the thick red E curve. The red horizontal lines show 10 cfu and 100 cfu thresholds for assessing protection from epithelial infection. 10^*x*^: the number on the x-axis should be used as the exponent of 10 to obtain the true value. Corresponding peak immune numbers are given in S3 Fig.

**Fig 10:**
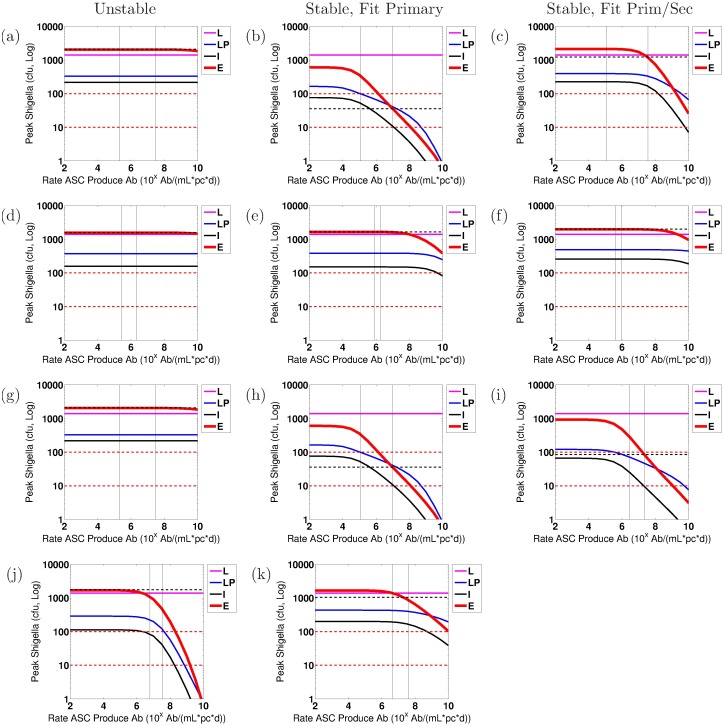
Predicted peak *Shigella* cfu levels when parameters *β*_*A*_ and *β*_*G*_ are varied together on a log scale from 10^2^ to 10^10^ (Ab/mL)/ASC/d. These control the rates at which ASC produce IgA and IgG, respectively. All other parameters are fixed to the best-fit parameter values given in Tables 3 and 4 for the model when fit to (a–c) EcSf2a-2 trials LPS data, (d–f) 2457T study LPS data, (g-i) EcSf2a-2 trials OMP data, (j–k) 2457T study OMP data. Model predictions for peak *Shigella* values by day 7 of infection are shown for the lumen (L), lamina propria (LP), engulfed inside innate immune cells (I), or in the epithelium (E). These are primary (a,d,g,j) and positive, stable secondary (b,e,h,k) infection dynamics fit to primary infection data only or are positive, stable secondary infection dynamics (c,f,i) resulting from fitting both primary and secondary infection data. Results with an unstable nontrivial equilibrium produced when fitting to both primary and secondary infection data are not shown due to strong similarity to the displayed unstable primary infection best-fit results. No best-fit parameter set for OMP measurements from the 2457T rechallenge study produced a positive stable nontrivial equilibrium when fitting both primary and secondary infection data. Vertical lines indicate best-fit parameter values. The horizontal black line shows the best-fit day 7 epithelial *Shigella* infection numbers for comparison to the thick red E curve. The red horizontal lines show 10 cfu and 100 cfu thresholds for assessing protection from epithelial infection. 10^*x*^: the number on the x-axis should be used as the exponent of 10 to obtain the true value. Corresponding peak immune numbers are given in S4 Fig.

**Fig 11:**
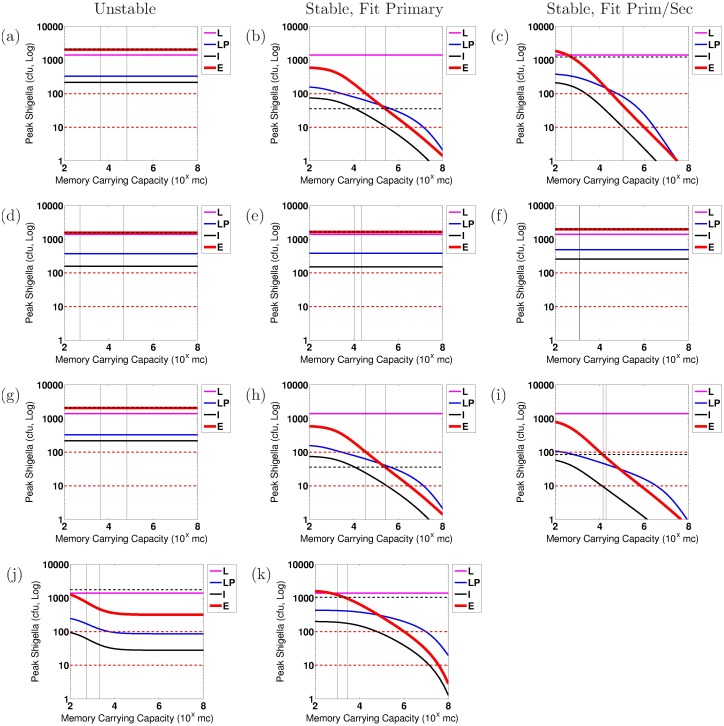
Predicted peak *Shigella* cfu levels when parameters *κ*_*A*_ and *κ*_*G*_ are varied together on a log scale from 10^2^ to 10^8^ B_M_. These are the carrying capacities of IgA-type and IgG-type memory B cells, respectively. All other parameters are fixed to the best-fit parameter values given in Tables 3 and 4 for the model when fit to (a–c) EcSf2a-2 trials LPS data, (d–f) 2457T study LPS data, (g-i) EcSf2a-2 trials OMP data, (j–k) 2457T study OMP data. Model predictions for peak *Shigella* values by day 7 of infection are shown for the lumen (L), lamina propria (LP), engulfed inside innate immune cells (I), or in the epithelium (E). These are primary (a,d,g,j) and positive, stable secondary (b,e,h,k) infection dynamics fit to primary infection data only or are positive, stable secondary infection dynamics (c,f,i) resulting from fitting both primary and secondary infection data. Results with an unstable nontrivial equilibrium produced when fitting to both primary and secondary infection data are not shown due to strong similarity to the displayed unstable primary infection best-fit results. No best-fit parameter set for OMP measurements from the 2457T rechallenge study produced a positive stable nontrivial equilibrium when fitting both primary and secondary infection data. Vertical lines indicate best-fit parameter values. The horizontal black line shows the best-fit day 7 epithelial *Shigella* infection numbers for comparison to the thick red E curve. The red horizontal lines show 10 cfu and 100 cfu thresholds for assessing protection from epithelial infection. 10^*x*^: the number on the x-axis should be used as the exponent of 10 to obtain the true value. Corresponding peak immune numbers are given in S5 Fig.

To investigate other potential immune targets, we carry out numerical sensitivity analysis on our model and plot key results in Figs 8–11 with corresponding predicted immune levels in S2–S5 Figs. We parameterize our model with the best-fit parameterizations in eight different cases each for EcSf2a-2 and 2457T data: fit to the primary infection only and either producing a stable or unstable nontrivial equilibrium; and fit to both the primary and secondary infection while the nontrivial equilibrium is either stable or unstable. From each of these best-fit tuples for LPS and separately for OMP, we widely vary one parameter at a time and search for which, if any, can reduce the epithelial *Shigella* load to below a threshold of 10 cfu on day 7 of a secondary infection. No best-fit parameterizations gave epithelial *Shigella* values below 10 cfu on day 7, while three EcSf2a-2 best-fit parameterizations (Panels b, h, and i in Figs 8–11) gave values already below a weaker threshold of 100 cfu. Importantly, the true threshold for an asymptomatic host is unknown, so it is possible individuals could be symptomatic with epithelial bacteria below even a 10 cfu threshold; however, establishing a feasible threshold enables us to quantitatively compare cases.

We find that only eight (or four groups) of all of our 33 parameters can sufficiently limit the peak epithelial infection to below 10 cfu under one or more parameterizations. Specifically, we see the epithelial infection controlled by drastically reducing the rates that *Shigella* migrates into the lamina propria (*μ*_*I*_) or epithelium (*μ*_*E*_); substantially increasing the rate that B_M_ differentiate into ASC (*ξ*_1_ or *ξ*_2_); significantly increasing the rate at which antibodies are produced by activated ASC (*β*_*A*_ or *β*_*G*_); or substantially increasing the *Shigella*-specific B_M_ carrying capacity (*κ*_*A*_ or *κ*_*G*_), which is the number of *Shigella*-specific B_M_ at homeostasis between infections. These can be seen in Figs 8–11, which plot peak bacterial levels on day 7 of a secondary infection (following vaccination or infection). The best-fit parameter values in each instance are shown with vertical lines, with actual values given in Tables 3 and 4. We compare the epithelial *Shigella* curve (solid red) with 10 and 100 cfu thresholds (dashed red lines). The corresponding day 7 peak immune levels of Ab/mL, ASC, and B_M_ are given in S2–S5 Figs. Each group of parameters is varied together; for instance, the listed migration rates (*μ* terms) are kept identical to one another. All other parameters show little-to-no impact on peak epithelial *Shigella* numbers and never drop below the 10 cfu epithelial infection threshold despite widely varying their values, sometimes beyond the range of the previous Latin hypercube sampling. Those that drop below the 100 cfu threshold are discussed at the end. Notably, neither Υ (the B_M_ symmetric vs asymmetric division percentage added to this model) nor the length of the incubation period time delay before naive activation affect the degree of epithelial infection (not shown because flat).

The *Shigella* migration rates to which the model dynamics are sensitive (*μ*_*E*_ and *μ*_*I*_) must be reduced to very small values (Fig 8) to limit the bacterial load within the epithelium; such a reduction would essentially stop the *Shigella* infection before it starts. However, this would require an immune mechanism different from anti-LPS or -OMP antibody targeting, which is already represented by other terms in the model. Since the causative immune agent is not the currently modeled immune response against LPS or OMP, anti-LPS and -OMP immune levels are nearly flat in almost all cases in S2 Fig despite the potential protection indicated in Fig 8.

Notably, the LPS response measured during the wt 2457T rechallenge study is predicted by the model to be insensitive to all parameter variations except a large drop in *Shigella* migration rates. This indicates that for a wt infection, an anti-LPS antibody response is only effective if it can almost completely block *Shigella* migration across or into the epithelium (and by a mechanism different than the modeled antibody blocking); otherwise, the model predicts that anti-LPS antibodies are not sufficient for disease prevention by any other mechanism even at much larger Ab/mL, ASC, or B_M_ levels. However, the same is not true for anti-OMP responses or for LPS responses during the EcSf2a-2 vaccine trials.

Epithelial infection levels are sensitive to the *ξ*_1_, *ξ*_2_ and *β*_*A*_, *β*_*G*_ parameter groups in all stable nontrivial anti-OMP cases (Panels h, i, and k in the following: Fig 9 with immune levels in S3 Fig for *ξ* terms, and Fig 10 and S4 Fig for *β* terms) and in some but not all anti-LPS responses (Panels b and c in Fig 9 and S3 Fig, and Panel c in Fig 10 and S4 Fig). These parameters are rates at which antibodies and ASC are created, respectively. Oddly, possible protection is observed even in cases without a stable immune state when fitting OMP measurements from the wt 2457T study and varying the *β* or *ξ* groups (Panel g in Figs 9 and 10), which suggests large pulses of anti-OMP Ab or ASC production even without vaccination might aid the prevention of symptoms in wt cases. However, this is not observed for LPS or with the EcSf2a-2 vaccine study OMP data. Comparing parameterized values to parameter zones that keep epithelial bacteria below the 10 cfu threshold in all sensitive cases reveals that the production rates of antibodies or ASC must be orders-of-magnitude faster than the best-fit values. Specifically, when *ξ*_1_ and *ξ*_2_, the rates at which B_M_ produce ASC, are varied, sufficient epithelial protection following the establishment of a stable immune equilibrium occurs with anti-OMP ASC numbers above 10^7^ and often greater than 10^9^ ASC, with correspondingly high antibody levels at or above 10^15^ Ab/mL. However, similar anti-OMP ASC and Ab levels are not protective with an anti-LPS immune response (Panels e and f in Fig 9 and S3 Fig). Varying *β*_*A*_ and *β*_*G*_, the rates at which ASC produce Ab, also shows that epithelial protection can occur, but is not guaranteed, when Ab levels rise above approximately 10^15^ Ab/mL. Here, a rise in ASC is not required for high Ab levels or protection; in fact, ASC numbers drop in all predicted protective cases as the rate at which ASC produce Ab increases because fewer ASC are required to sustain higher Ab levels.

In some, but not all, best parameter fits shown in Fig 11 and S5 Fig, substantially altering the B_M_ carrying capacities (*κ*_*A*_ and *κ*_*G*_) could reduce or even prevent the epithelial infection; that is, establishing a larger permanent pool of B_M_ could generate protective immunity. This sensitivity is observed when a stable nontrivial equilibrium is established in the EcSf2a-2 vaccine trial as well as for anti-OMP B_M_ in a 2457T wt rechallenge (Panels b, c, h, i, and k in Fig 11). The effect is strongest when the carrying capacity for B_M_ is increased from the best-fit values to establish lasting B_M_ populations of at least 10^6^ cells. However, such B_M_ numbers were not reached during infection in the 2457T LPS best-fit case despite raising the B_M_ carrying capacity well above 10^6^ cells. This suggests that establishing a larger permanent anti-OMP or anti-LPS B_M_ population above the current levels established with the EcSf2a-2 vaccine candidate or a wt infection can (but does not necessarily) raise overall B_M_ numbers and could correlate with protection in some, but not all, cases.

Comparison of Figs 8–11 in relation to the baseline parameterization of each (vertical lines and Tables 3 and 4) reiterates that all parameterizations that best fit the clinical data have parameters in, and often well into, the zones that produce epithelial infection levels above the 10 cfu threshold, as previously seen in Figs 4–7. If these parameter values can be raised biologically, epithelial invasion by *Shigella* can be reduced and even potentially entirely prevented. For instance, the epithelial *Shigella* levels in Panel i of Figs 8–11 drop below 1 cfu if any one of the four sensitive parameter groups is substantially altered. This suggests there might be multiple avenues by which to raise anti-OMP immunity to fully protective levels for the EcSf2a-2 vaccine. In contrast, altering wt 2457T anti-LPS immunity is predicted as never fully protective and wt anti-OMP immunity is fully protective solely when accompanied by a rise in *ξ*_1_ and *ξ*_2_, the rates at which B_M_ generate ASC. While we change only one parameter group at a time, an unexplored combination of parameter changes might also result in sufficient protection.

If we relax the protection threshold to 100 cfu on day 7, we have noted that three EcSf2a-2 vaccine best-fit parameterizations already are below this. These are the best-fit parameterizations for the EcSf2a-2 study that produced a stable equilibrium when fitting only the primary infection LPS data, only primary infection OMP data, or both primary and secondary infection OMP data. Yet we know the EcSf2a-2 vaccine was not on average protective at a group level, and thus 100 cfu is likely too weak a threshold for protection. Nevertheless, we use this 100 cfu threshold to describe how results change when the threshold considered is raised from 10 cfu. For the 10 cfu threshold, epithelial infection dynamics were sensitive to four parameter groups; for a 100 cfu threshold, the epithelial infection on day 7 can be lowered by changing one of 10 parameter groups. However, this is better parsed by separating the three cases already below threshold (Panels b, h, and i in Figs 8–11) from those which drop newly below threshold given a parameter change. The latter is rare and only occurs for a wt 2457T infection with an unstable equilibrium (no lasting immunity). We see that this case, Panel j in Figs 8–11, has a day 7 epithelial infection that drops below 100 cfu by altering any of the 4 previously sensitive parameter groups or by altering one of 3 new parameter groups as follows (not shown): raising the B_M_ growth rate (*ρ*), substantially decreasing the percent of B_M_ that act like memory stem cells (Υ), or substantially lowering natural antibody death rates (*δ*_*AE*_, *δ*_*AI*_, and *δ*_*G*_). It must be noted, however, that altering these parameters does not affect wt 2457T dynamics when a stable nontrivial immune equilibrium is established, and thus this sensitivity is not robust under multiple best-fit parameterizations. It is also interesting to look at which parameters exhibit sensitivity to parameter values for the three cases already below 100 cfu with their best-fit parameterizations (panels bhi). The epithelial *Shigella* numbers are entirely flat for most parameter values, indicating complete insensitivity of the epithelial infection to those parameters. The parameters to which the epithelial *Shigella* number show sensitivity in these three cases are the 4 previously sensitive parameter groups plus the following 4 parameter groups (not shown): the antigen-dependent B_M_ differentiation rates, *ϕ*_2*A*_ and *ϕ*_2*G*_; the antigen-independent B_M_ differentiation rate, *ϕ*_1_; the B_M_ growth rate, *ρ*; and the neutralization rates of *Shigella* by Ab, *α* and *γ*. The existence of these indicates that if a vaccine can establish sufficient anti-LPS or anti-OMP immunity to drop epithelial *Shigella* values below 100 cfu, additional immune parameters might gain feasibility as secondary immune targets and as conditional immune correlates.

## Discussion

In this paper, we build upon our original mathematical model [29] of the humoral immune response against *Shigella* by introducing asymmetric division of B_M_, removing nonessential delays, and comprehensively parameterizing the model with clinical anti-LPS and anti-OMP humoral immune data from three human *Shigella* studies in the 1990s: two EcSf2a-2 vaccine trials and a subsequent wt 2457T rechallenge study [30–32, 34]. We use Monte Carlo runs of Latin hypercube sampling with least-squares fitting to parameterize the model both against only primary infection data and against primary and secondary infection data simultaneously for each study and each immune target in order to explore which observed trends are parameterization-dependent versus common across parameterizations. To avoid the pitfalls of overfitting a large number of parameters to data, we do not seek to optimize every parameter value individually but rather consider a large number of best-fit parameter tuples as a whole and identify trends across many best-fit tuples. Our modeling results suggest that, on average, humans would be symptomatic with shigellosis following a purely anti-LPS or -OMP humoral immune response due to an uncontrolled infection of gut epithelial cells that is present across all best-fit model parameterizations. We conduct identifiability analysis to gain insight on which parameter values can be uniquely determined, and we use sensitivity analysis to explore which model parameter values must be altered to prevent epithelial invasion and destruction by *Shigella* bacteria. We identify four key parameter groups as potential vaccine targets or immune correlates.

Results presented in this paper are only as accurate as the mathematical model, and all models are approximations. Limitations of our approach include utilization of serum IgA as an indirect measure of luminal IgA as well as assessment of immune markers that correlate with protection at the group rather than individual level. Predicted levels of epithelial invasion could be underestimates due to not directly modeling cell-to-cell spread of *Shigella* within the epithelium, which could exacerbate epithelial bacteria levels even above model predictions. Furthermore, immunological thresholds for protection are not clinically known and thus achieving thresholds set in this paper does not guarantee protection from disease. These limitations are transparently considered in the conclusions made and may be addressed in future studies. Modeling results and predictions should be confirmed with biological studies before being clinically employed. Biological validation of these results is difficult due to limited animal models for *Shigella* and the necessity for human clinical work. However, a strength of mathematical modeling is the ability to first explore conditions theoretically that are difficult to examine biologically and thereby to inform future biological investigation.

Our best-fit parameterizations that match the EcSf2a-2 vaccine trial data reveal that the vaccine likely established some immunity in some individuals (although not protective at the group level), which makes sense given the 27-36% efficacy of the trials [30–32, 34]. That is, while the EcSf2a-2 vaccine candidate was not sufficiently efficacious at a group level, the model suggests that measured humoral immune levels during the post-vaccine wt challenge are only possible if some lasting anti-LPS and anti-OMP immunity was established. Nevertheless, the epithelial bacterial numbers (the purple dashed *S*_*C*_ curves) in Figs 4 and 6, and Table 5 indicate that hosts vaccinated with an EcSf2a-2 vaccine would still be symptomatic with these established levels of immunity if 100 cfu of bacteria on day 7 is an insufficient threshold for protection (which is suggested by the 27-36% efficacy).

While our modeling suggests the EcSf2a-2 vaccine established some levels of lasting LPS and OMP immunity, the model results also suggest that, to reproduce wt data, little-to-no lasting anti-LPS or -OMP humoral immunity is established by a primary wt 2457T infection. That is, the model matches data best when there is no stable nontrivial equilibrium or when the nontrivial equilibrium has very few Ab, ASC and B_M_. Since it is known that humans can develop immunity to wt *Shigella* infections, these modeling results indicate that the primary immune factors responsible for conferring immunity are likely not (or not solely) anti-LPS or anti-OMP humoral immune responses.

Thus, in what is perhaps our strongest modeling result, we observe that in every best-fit model parameterization using any primary and secondary infection data, bacteria invade the epithelium and rise quickly and uncontrollably (Figs 4–7). That is, under the 10 cfu day 7 epithelial infection threshold, the model predicts epithelial escape of the bacteria in every best-fit case regardless of parameterization, bacterial target (LPS or OMP), or clinical study (EcSf2a-2 or 2457T). Since it is the epithelial infection that induces the worst symptoms of shigellosis [50], this uncontrolled epithelial expansion indicates that our model’s hypothetical patient (and the average EcSf2a-2 or 2457T study volunteer when protected solely by an anti-LPS or -OMP humoral response) is symptomatic to heavily symptomatic. Thus, our modeling results indicate that a vaccine or natural immunity targeting only LPS and/or OMP will not be protective against *Shigella*. We amend this slightly after conducting sensitivity analysis to determine whether altering individual immune rates from their parameterized values can increase the effectiveness of an LPS- or OMP-targeted vaccine. Nevertheless, any suggested alterations do not fit observed wild-type or vaccine trial measurements, and it is not yet known if they are biologically feasible.

Putting the ubiquitous rise in epithelial infection levels across all best-fit parameterizations together with the observation that some clinical trial and rechallenge study participants were protected at the measured levels of anti-LPS and -OMP antibodies suggests that healthy individuals were protected at least in part by immune components other than anti-LPS and anti-OMP humoral immune responses. We are unable with these data to determine what those might be, but we use sensitivity analysis to explore possibilities. Our numerical sensitivity analysis reveals that severe epithelial infection is insensitive to all but four parameter groups. That is, limiting the epithelial infection to 10 cfu by day 7 of infection requires radically lowering the rates that *Shigella* migrates into the lamina propria (*μ*_*I*_) or epithelium (*μ*_*E*_); markedly increasing the rate that B_M_ differentiate into ASC (*ξ*_1_ or *ξ*_2_); substantially increasing the rate at which antibodies are produced by activated ASC (*β*_*A*_ or *β*_*G*_); or significantly increasing the *Shigella*-specific B_M_ carrying capacity (*κ*_*A*_ or *κ*_*G*_), that is, the homeostatic *Shigella*-specific B_M_ population size. Limiting the epithelial infection to below 100 cfu is less difficult, and we also explore the broader set of parameter targets by which to do this; however, those parameter alterations might not be sufficiently protective. As a caveat, the 10 cfu and 100 cfu thresholds for the epithelial infection are the numbers of bacteria in the epithelium that escape all immune action; thus, in future models that include CMI, collaboration between humoral and cellular compartments could provide protection even with more bacteria present.

Decreasing the migration of *Shigella* from the lumen (that is, lowering *μ*_*E*_) to prevent *Shigella* infections from beginning to establish would require, for instance, secretory IgA antibodies that block *Shigella* entry into the lamina propria via M cells. However, decreased migration from the lumen would require a mode of action by immune components that is different than the modeled mass-action removal of bacteria by LPS- or OMP-directed IgA antibodies, which is not sufficient to prevent infection. On the other hand, lowering the migration rate of *Shigella* from the lamina propria into the epithelium (that is, *μ*_*I*_) might be accomplished with antibodies that specifically target the epithelial entry proteins displayed by *Shigella* for brief periods of time. Such proteins include IpaB/C/D/A, IpgC/D/E, and other known bacterial products [63]. We hope to explore this more in future modeling work that takes into account the short exposure times and brief window for antibody targeting of these proteins. Additionally, measuring secretory IgA in future studies rather than utilizing serum IgA as an indirect estimate will help clarify this issue.

Two parameter groups (*ξ*_1_, *ξ*_2_ and *β*_*A*_, *β*_*G*_) to which the epithelial infection levels are sensitive in most stable nontrivial cases control the rates at which antibodies and ASC are created. Orders-of-magnitude faster creation of antibodies or their secreting cells is required for preventing an epithelial infection. This faster production rate maintains total antibody levels at those observed in the data starting from often small pools of B_M_. It should be noted that we change only one parameter group at a time, and thus smaller increases in combinations of B_M_, ASC, and antibodies might have similar effects; this possibility was partially explored via the parameter fitting process and did not produce a best-fitting parameter combination within the tuples investigated. The observation from Panel j in Figs 9 and 10 that a substantial decrease in epithelial bacteria might be possible even without a stable immune state by varying the *β* or *ξ* groups suggests that mimicking high production rates by prophylactically dosing individuals with large amounts of antibodies targeting OMP, even without creating long-term immunity, could be effective. However, this was observed only when fitting OMP measurements from the 2457T rechallenge study and thus is heavily study- and parameterization-dependent. For vaccine design purposes, more attention should perhaps be paid to the stable nontrivial equilibrium cases that suggest orders-of-magnitude increases in the rates that antibodies (both IgA and IgG) or ASC are produced above all current best-fit levels would be required to keep the epithelial infection below 10 cfu on day 7. It is not known if lowering the epithelial *Shigella* peak below this dose will be sufficient for protection.

Significantly raising the B_M_ carrying capacities (*κ*_*A*_ and *κ*_*G*_) could limit the epithelial infection in some, but not all, cases. This suggests that establishing a larger permanent pool of B_M_ via vaccination could prevent a severe epithelial infection, if this boosting sufficiently raises the B_M_ carrying capacity in addition to the transitory number of B_M_. In fact, Panels c and i in Fig 11 predict that the epithelial infection could be almost entirely prevented if the long-term levels of B_M_ directed at OMP could be raised by 2–4 orders of magnitude; this is purely theoretical and remains to be explored experimentally. However, the lack of sensitivity of epithelial bacterial numbers to the LPS B_M_ carrying capacity in the 2457T rechallenge study (Panels d–f in Fig 11) suggests that establishing a larger B_M_ permanent pool will not be sufficient for protection in all cases. Broader protection across all cases is obtained solely by lowering *Shigella* migration rates into the lamina propria and epithelium.

Sensitivity analysis suggests that creating better antibodies through somatic hypermutation and/or affinity maturation might not be protective, since severe epithelial infection dynamics are insensitive to the parameters that relate to antibody effectiveness (*α* and *γ*, not shown). Thus, refining the ability of anti-LPS and anti-OMP antibodies to find, be tuned to, and eliminate *Shigella* is predicted by modeling to not sufficiently protect against epithelial invasion. Furthermore, comparing requirements to meet the 100 cfu threshold versus the 10 cfu threshold for the day 7 epithelial infection reveals some potential immune correlates that are conditional upon establishing EcSf2a-2 vaccine levels of anti-LPS and anti-OMP humoral immunity. That is, while the unconditionally sensitive immune parameters established by the model are the rate that *Shigella* migrates into the lamina propria or epithelium, the rate that B_M_ differentiate into ASC, the rate at which antibodies are produced by activated ASC, and the *Shigella*-specific B_M_ carrying capacity, additional conditional targets for enhancing an already partially protective vaccine include the B_M_ differentiation rates, the B_M_ growth rate, and the neutralization rates of *Shigella* by Ab. This underscores the idea that multiple immune mechanisms and bacterial targets might work in combination to sufficiently protect against *Shigella* infections.

In moving forward, modeling conclusions should be tested and confirmed clinically. A planned future modeling direction is to examine *Shigella* immune protection at the individual rather than group level; that is, we hope to utilize our model to discern immunological differences between those individuals who were protected by the EcSf2a-2 vaccine or wild-type challenge versus those who were not. Additionally, we plan to track epithelial cell damage more overtly in the model in order to directly integrate or predict clinical disease outcomes for each individual. Future modeling should also include new immune targets and correlates that become known in the literature. Measuring and modeling CMI and spatially distinct peripheral versus mucosal immune responses may also assist in a better understanding of the protective immune response against *Shigella*.

## Supporting information

S1 Fig The best-fit parameter tuples resulting from every grand run plotted together. These best-fit parameter values result from fitting the model to every combination of the following four characteristics: EcSf2a-2 or 2457T study, LPS or OMP, fitting primary data (1) or fitting both primary and secondary data (2), and producing a stable or unstable nontrivial equilibrium. The parameters are listed on the horizontal axis in the same order as Table 1. The 3.5-day incubation period delay is included. The parameter tuples are listed in Tables 3 and 4. Black lines show the maximum and minimum of the region explored by Latin Hypercube sampling. The entire space was searched but only the best-fit parameter tuple for each scenario is displayed. The colors are as follows. Magenta: stable, fit (1); Cyan: unstable, fit (1); Red: stable, fit (2); Blue: unstable, fit (2). Stable (some immunity) versus unstable (no immunity) parameter values have been slightly offset horizontally to make them easier to distinguish. Each parameter tuple as a whole is optimized, not each individual parameter.
(EPS)

S2 Fig Predicted peak immune levels when parameters *μ*_*E*_ and *μ*_*I*_ are varied together from 0.01 to 1/d. These control the rate at which *Shigella* migrates from the lumen into the lamina propria (LP) or from the LP into the epithelium. All other parameters are fixed to the best-fit parameter values given in Tables 3 and 4 for the model when fit to (a–c) EcSf2a-2 trials LPS data, (d–f) 2457T study LPS data, (g-i) EcSf2a-2 trials OMP data, (j–k) 2457T study OMP data. Model predictions for peak immune values by day 7 for Ab/mL, ASC, and B_M_ are shown. These are primary (a,d,g,j) and positive, stable secondary (b,e,h,k) infection dynamics fit to primary infection data only or are positive, stable secondary infection dynamics (c,f,i) resulting from fitting both primary and secondary infection data. Results with an unstable nontrivial equilibrium produced when fitting to both primary and secondary infection data are not shown due to strong similarity to the displayed unstable primary infection best-fit results. No best-fit parameter set for OMP measurements from the 2457T rechallenge study produced a positive stable nontrivial equilibrium when fitting both primary and secondary infection data. Vertical lines indicate best-fit parameter values. Corresponding peak *Shigella* numbers are given in Fig 8.
(EPS)

S3 Fig Predicted peak immune levels when parameters *ξ*_1_ and *ξ*_2_ are varied together on a log scale from 10^0^ to 10^6^ ASC/B_M_. These control the rates at which new ASC are generated by B_M_ in the absence or presence of antigenic stimulation, respectively. All other parameters are fixed to the best-fit parameter values given in Tables 3 and 4 for the model when fit to (a–c) EcSf2a-2 trials LPS data, (d–f) 2457T study LPS data, (g-i) EcSf2a-2 trials OMP data, (j–k) 2457T study OMP data. Model predictions for peak Ab/mL, ASC, and B_M_ values by day 7 are shown. These are primary (a,d,g,j) and positive, stable secondary (b,e,h,k) infection dynamics fit to primary infection data only or are positive, stable secondary infection dynamics (c,f,i) resulting from fitting both primary and secondary infection data. Results with an unstable nontrivial equilibrium produced when fitting to both primary and secondary infection data are not shown due to strong similarity to the displayed unstable primary infection best-fit results. No best-fit parameter set for OMP measurements from the 2457T rechallenge study produced a positive stable nontrivial equilibrium when fitting both primary and secondary infection data. Vertical lines indicate best-fit parameter values. 10^*x*^: the number on the x-axis should be used as the exponent of 10 to obtain the true value. Corresponding peak *Shigella* numbers are given in Fig 9.
(EPS)

S4 Fig Predicted peak immune levels when parameters *β*_*A*_ and *β*_*G*_ are varied together on a log scale from 10^2^ to 10^10^ (Ab/mL)/ASC/d. These control the rates at which ASC produce IgA and IgG, respectively. All other parameters are fixed to the best-fit parameter values given in Tables 3 and 4 for the model when fit to (a–c) EcSf2a-2 trials LPS data, (d–f) 2457T study LPS data, (g-i) EcSf2a-2 trials OMP data, (j–k) 2457T study OMP data. Model predictions for peak Ab/mL, ASC, and B_M_ values by day 7 are shown. These are primary (a,d,g,j) and positive, stable secondary (b,e,h,k) infection dynamics fit to primary infection data only or are positive, stable secondary infection dynamics (c,f,i) resulting from fitting both primary and secondary infection data. Results with an unstable nontrivial equilibrium produced when fitting to both primary and secondary infection data are not shown due to strong similarity to the displayed unstable primary infection best-fit results. No best-fit parameter set for OMP measurements from the 2457T rechallenge study produced a positive stable nontrivial equilibrium when fitting both primary and secondary infection data. Vertical lines indicate best-fit parameter values. 10^*x*^: the number on the x-axis should be used as the exponent of 10 to obtain the true value. Corresponding peak *Shigella* numbers are given in Fig 10.
(EPS)

S5 Fig Predicted peak immune levels when parameters *κ*_*A*_ and *κ*_*G*_ are varied together on a log scale from 10^2^ to 10^8^ B_M_. These are the carrying capacities of IgA-type and IgG-type memory B cells, respectively. All other parameters are fixed to the best-fit parameter values given in Tables 3 and 4 for the model when fit to (a–c) EcSf2a-2 trials LPS data, (d–f) 2457T study LPS data, (g-i) EcSf2a-2 trials OMP data, (j–k) 2457T study OMP data. Model predictions for peak Ab/mL, ASC, and B_M_ values by day 7 are shown. These are primary (a,d,g,j) and positive, stable secondary (b,e,h,k) infection dynamics fit to primary infection data only or are positive, stable secondary infection dynamics (c,f,i) resulting from fitting both primary and secondary infection data. Results with an unstable nontrivial equilibrium produced when fitting to both primary and secondary infection data are not shown due to strong similarity to the displayed unstable primary infection best-fit results. No best-fit parameter set for OMP measurements from the 2457T rechallenge study produced a positive stable nontrivial equilibrium when fitting both primary and secondary infection data. Vertical lines indicate best-fit parameter values. 10^*x*^: the number on the x-axis should be used as the exponent of 10 to obtain the true value. Corresponding peak *Shigella* numbers are given in Fig 11.
(EPS)
