# Correction: A clinically parameterized mathematical model of *Shigella* immunity to inform vaccine design

**DOI:** 10.1371/journal.pone.0196367

**Published:** 2018-04-19

**Authors:** Courtney L. Davis, Rezwanul Wahid, Franklin R. Toapanta, Jakub K. Simon, Marcelo B. Sztein

Typesetting errors introduced horizontal lines in Table 2. The corrected Table 2 is included here as a Supporting Information file.

## Supporting information

S6 File **Table 2. Nontrivial disease-free equilibrium values.** Values of model state variables at the nontrivial disease-free equilibrium for which both IgA and IgG are present are given for the EcSf2a-2 trials or 2457T rechallenge study data. For each, the model is parameterized with data fit to either primary infection data alone or to both primary and secondary infection data. The positive, stable nontrivial equilibrium values are given, representing the long-term presence of some immunity, while in the unstable cases, the trivial equilibrium is instead approached. No positive nontrivial equilibrium was found for parameters that best fit the OMP measurements for the 2457T rechallenge study. The nontrivial equilibrium is evaluated at the corresponding parameter values given in the other tables. The equations for this nontrivial equilibrium are also given, as well as eigenvalues of the Jacobian for the linearized model. The equilibria are all positive and the eigenvalues are all negative if the positivity and stability conditions given in the text are met. These are sufficient, and for positivity necessary, conditions.
(PDF)
