# Supplementary material for: Correction: A clinically parameterized mathematical model of Shigella immunity to inform vaccine design
Source: PLoS One. 2018 Apr 19;13(4):e0196367. doi: 10.1371/journal.pone.0196367 (PMC5908090; doi:10.1371/journal.pone.0196367)
Supplement: S6 File — Table 2. Nontrivial disease-free equilibrium values. Values of model state variables at the nontrivial disease-free equilibrium for which both IgA and IgG are present are given for the EcSf2a-2 trials or 2457T rechallenge study data. For each, the model is parameterized with data fit to either primary infection data alone or to both primary and secondary infection data. The positive, stable nontrivial equilibrium values are given, representing the long-term presence of some immunity, while in the unstable cases, the trivial equilibrium is instead approached. No positive nontrivial equilibrium was found for parameters that best fit the OMP measurements for the 2457T rechallenge study. The nontrivial equilibrium is evaluated at the corresponding parameter values given in the other tables. The equations for this nontrivial equilibrium are also given, as well as eigenvalues of the Jacobian for the linearized model. The equilibria are all positive and the eigenvalues are all negative if the positivity and stability conditions given in the text are met. These are sufficient, and for positivity necessary, conditions. (PDF) [file pone.0196367.s001.pdf]

Table 2. Nontrivial Disease-Free Equilibrium Values

	S_E, S_{I1}, S_N, S_{I2}, S_C cfu	A_E Ab/mL	A_I Ab/mL	G Ab/mL	P_A, P_G Cells	M_A, M_G Cells
EcSf2a-2 LPS Study	0	3.1×10^9	3.5×10^9	2.4×10^{12}	$9,610$	$20,050$
Fit Primary Data					$75,146$	$156,780$
2457T LPS Study	0	5.1×10^{10}	1.2×10^{11}	5.6×10^{11}	$15,685$	$19,957$
Fit Primary Data					$7,647$	$9,730$
EcSf2a-2 OMP Study	0	3.1×10^9	3.5×10^9	2.4×10^{12}	$9,610$	$20,050$
Fit Primary Data					$75,146$	$156,780$
2457T OMP Study	0	2.1×10^9	4.7×10^8	2.7×10^{11}	24	933
Fit Primary Data					69	$2,668$
EcSf2a-2 LPS Study	0	6.0×10^{10}	1.4×10^{11}	2.2×10^{11}	$611,620$	$88,311$
Fit Primary and					$3,040$	439
Secondary Data						
2457T LPS Study	0	2.0×10^7	1.3×10^7	1.6×10^8	4	73
Fit Primary and					4	71
Secondary Data						
EcSf2a-2 OMP Study	0	1.8×10^{11}	3.5×10^{10}	1.7×10^{13}	$75,926$	$8,585$
Fit Primary and					$196,870$	$22,261$
Secondary Data						

Nonzero Terms

$$A_E = \frac{\omega}{\delta_{AE}} A_I \qquad P_A = \frac{\Upsilon^* \xi_1 \phi_1}{\delta_{PA}} M_A \qquad M_A = \frac{\kappa_A(\rho - \Upsilon\phi_1)}{\rho}$$

$$A_I = \frac{\beta_A}{\delta_{AI} + \omega} P_A \qquad P_G = \frac{\Upsilon^* \xi_1 \phi_1}{\delta_{PG}} M_G \qquad M_G = \frac{\kappa_G(\rho - \Upsilon\phi_1)}{\rho}$$

$$G = \frac{\beta_G}{\delta_G} P_G$$

Eigenvalues of Jacobian at Nontrivial Equilibrium

$$-\delta_{PG} \qquad \sigma_N - \delta_{SN} - \mu_N \qquad -\delta_{SI} - \mu_I - \frac{\gamma\beta_G\xi_1\phi_1\kappa_G}{\delta_G\delta_{PG}\rho}\Upsilon^*(\rho - \Upsilon\phi_1)$$

$$-\delta_G \qquad -(\rho - \Upsilon\phi_1) \qquad -\delta_{SI} - \mu_I - \mu_{NI} - \frac{\gamma\beta_G\xi_1\phi_1\kappa_G}{\delta_G\delta_{PG}\rho}\Upsilon^*(\rho - \Upsilon\phi_1)$$

$$\delta_{PA} \qquad -(\rho - \Upsilon\phi_1) \qquad -\delta_{SE} - \mu_E - \frac{\alpha\omega\beta_A\xi_1\phi_1\kappa_A}{\delta_A E(\delta_{AI} + \omega)\delta_{PA}\rho}\Upsilon^*(\rho - \Upsilon\phi_1)$$

$$\delta_{AE} \qquad \delta_{AI} - \omega$$

Values of model state variables at the nontrivial disease-free equilibrium for which both IgA and IgG are present are given for the EcSf2a-2 trials or 2457T rechallenge study data. For each, the model is parameterized with data fit to either primary infection data alone or to both primary and secondary infection data. The positive, stable nontrivial equilibrium values are given, representing the long-term presence of some immunity, while in the unstable cases, the trivial equilibrium is instead approached. No positive nontrivial equilibrium was found for parameters that best fit the OMP measurements for the 2457T rechallenge study. The nontrivial equilibrium is evaluated at the corresponding parameter values given in the other tables. The equations for this nontrivial equilibrium are also given, as well as eigenvalues of the Jacobian for the linearized model. The equilibria are all positive and the eigenvalues are all negative if the positivity and stability conditions given in the text are met. These are sufficient, and for positivity necessary, conditions.
